# Drivers of migrant passerine composition at stopover islands in the western Mediterranean

**DOI:** 10.1038/s41598-022-06912-2

**Published:** 2022-02-21

**Authors:** Germán M. López-Iborra, Antonio Bañuls, Joan Castany, Raül Escandell, Ángel Sallent, Manuel Suárez

**Affiliations:** 1 Departamento de Ecología/IMEM Ramon Margalef, Universidad de Alicante, Alicante, Spain; 2 Grupo Local SEO-Alicante, SEO/BirdLife, Alicante, Spain; 3 Grup Au d’Ornitologia, Castelló, Spain; 4 Societat Ornitològica de Menorca, Ap. de correus 83, 07720 Es Castell, Spain; 5 Asociación de Naturalistas del Sureste, Murcia, Spain; 6 Grup Balear d’Ornitologia i Defensa de La Naturalesa (GOB), Palma de Mallorca, Spain

**Keywords:** Animal migration, Biodiversity, Ecology, Biogeography

## Abstract

Clues used by migrant birds to select sites for stopover are much less known than their reasons for leaving. Habitat characteristics and geographical location may affect the decision to use an island as a stopover site in different ways for different species. Thus, abundance and composition of migrants may be expected to differ between islands. Using standardized ringing from 9 western Mediterranean islands we evaluate drivers of abundance of trans-Saharan migrant passerines, specifically the role of species continental abundance, island characteristics and geographical location. Although continental abundance is a main driver of migrant composition on all islands migrant composition differs between them. Redundancy analysis and species response models revealed that the main drivers were distance to the nearest land toward the south, which has a positive effect on the number of migrants of most species, and island area, which appears as an important cue used for selecting a stopover island. Species whose abundance is positively related to island area have more pointed wings while species affected by distance to land toward the south have relatively more rounded wings. This suggests a hypothesis on the mechanism that may generate differences in passerine migrant composition between islands based on better efficiency of more pointed wings for long-distance flight.

## Introduction

Most long-distance migrants breeding in the western Palearctic need to cross two large barriers in each migration, the Sahara Desert and the Mediterranean Sea. The Sahara may be crossed in a continuous flight^1^ or doing stopovers at appropriate sites^2^. When crossing the sea, the only possible stopover sites, apart from urgent stops on boats, are islands. In both cases, the characteristics of habitats at stopover sites may strongly influence the probability of success of the migratory trip^3^. Species composition and abundance of bird migrants at stopover sites may depend on factors operating at several scales. At a wide spatial scale, continental abundance of each migrant species directly determines the global number of migrants and might be a potential driver of the relative abundance of each species at stopover sites. However, different specific migration strategies or decision rules for stopover^4^ could cause the relative abundance of migrants to deviate from mirroring their continental abundance.

To optimize migratory success, migrant birds must take at least two types of important decisions: whether or not to stop at a potential stopover site appearing over the course of a trip and, once landed, when to leave the site^5,6^. There is a huge amount of research about the factors that influence the decision to leave^7^. These studies have identified several intrinsic factors, such as fuel load^8,9^ or refueling rate^6,10,11^, and extrinsic factors such as weather including wind^12^, among other factors. On the contrary, the clues used by migrants to select particular sites for stopover are much less known^5^. Several studies have identified some important variables for mainland stopover sites, such as habitat characteristics, in particular forest cover^8,13–15^, and meteorological factors, such as rain^16^, but such factors are far less understood for island stopover sites (see ^17^ for an exception including islands close to the coast). Furthermore, wing morphology is related to the efficiency and range of flight^18,19^ so that flight performance related to wing shape could influence travel time or distance when crossing wide barriers^20^ and therefore is expected to affect species responses to island characteristics.

**Figure 1:**
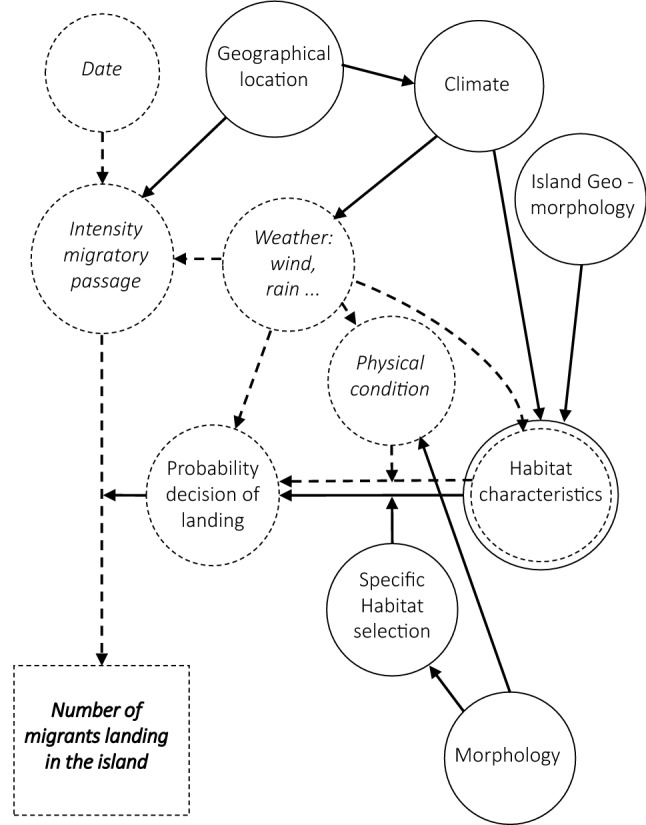
Conceptual model of the factors affecting the number of migrants of a species that make stopovers on an island. Continuous line circles and arrows identify factors that are constant over the long term. Dashed line circles and arrows identify factors that are highly variable, and change from day to day. Habitat characteristics are intermediate because some habitat components are stable (e.g. dominant vegetation types) while others may change through the migration season or between seasons (e.g. abundance of fruits or insects). Arrows ending over other arrows represent the modulating effect of some variables on the relationship of habitat characteristics and the landing decision. The interaction of the daily intensity of migratory passage and the probability of the decision to land of individual birds should determine the number of migrants actually landing each day. The sum of these numbers yields the number of migrants in a given season.

If habitat characteristics affect the decision to use a stopover site in different ways for different species, it may be expected that abundance and composition of migrants will differ between islands. The number and species composition of migrants that stop on islands in a given season would depend on a large diversity of variables. Some of these variables fluctuate greatly over time within a migratory season, such as daily intensity of the migratory passage, the weather along the migratory path and the physical conditions of migrants. For instance, migrants in poor condition may be more prone to landing on an island than healthier birds^5,21^ and unfavorable winds may promote the landing of more migrants^22^. The average distribution of these fluctuating variables depends on other island features that are more stable over time. Some of these features are related to the geographical location of the islands^17^, which determines its location relative to the main migration flyways, their distance to the mainland source of migrants, prevailing winds and, to a large extent, their climate. On the other hand, islands located in the same geographical region may differ in some specific characteristics that may also have an effect on migrant decisions to land. These include their size and topography, which are likely related to the diversity of habitats that a migrant may find, and to their vegetation cover. The interaction between the particular characteristics of an island, the specific habitat selection rules of the different migrant species and the physical condition of individuals should determine the probability of the decision to land (Fig. 1). However, the number of migrants actually landing would depend on the intensity of the migratory passage over the island on a given day. Therefore, stable and changing island features interact to determine the abundance and composition of the migrants that stop on a given island each day along a migratory passage, and consequently the number of migrants landing on an island is highly variable on a daily scale^23^.

The stable features of islands would determine in part the abundance and composition of migrants and, in the long term, we expect that a general pattern would emerge for each island if migration seasons of several years are analyzed. However, the effect of species continental abundance might still be detectable and contribute to making migrant composition similar between islands. Therefore, the first aim of this paper is to assess if the abundance distribution of trans-Saharan migrant passerines that make stopovers at western Mediterranean islands is determined by the continental abundance of the species. The second objective is to evaluate beta diversity of these migrants, to estimate how it is partitioned among spatial and temporal components and determine if there are differences in the composition of migrants between this set of islands. We focus on trans-Saharan passerines in spring migration because ringing campaigns on these islands are timed to detect the passage of this group of species and do not sufficiently cover the phenology of short-distance migrants.

The third objective of this paper is to identify stable island characteristics that determine the gradients in migrant species composition by using community ordination techniques. These gradients would be shaped by species-specific responses to island characteristics that would determine the abundance of each species at stopover sites. Therefore, the fourth objective of this paper is to identify drivers of migrant species abundance at these islands. To attain these two objectives, we evaluate the relative importance of variables related to the geographical location of the islands and variables related to the habitats existing in them. Within the former, we include the distance to large land masses (continents or large islands) that could act as a source of migrants or could affect the decision to land on an island. In spring migration, larger distances to sources of migrants situated south of the islands are expected to increase the number of birds likely to make stopovers, since they would have been flying for a longer period of time^1^. On the other hand, on islands that are close to land masses located in other, non-southerly directions it is possible that some of the migrants prefer to continue flying a short distance to arrive at larger pieces of land that may offer more resources. In these islands migrant abundance is expected to decrease with distance to these land masses. We also analyze the effect of the location of the island in the west–east gradient since migrant species differ in their main routes of migration, with some crossing the Mediterranean in a wide front while others are more concentrated on western or eastern routes^24^. Concerning the specific characteristics of the islands, we analyze the size and topography of the island, which are likely related to the diversity of habitats existing there, and the Normalized Difference Vegetation Index (NDVI), which is an indicator of productivity and is expected to be related to food resource abundance for landed birds^25^. Finally, since species responses to island characteristics may be affected by wing morphology, we explored if species whose abundance is related to the different island variables also differ in wing pointedness and aspect ratio.

## Materials and methods

### Study islands and bird data

**Figure 2:**
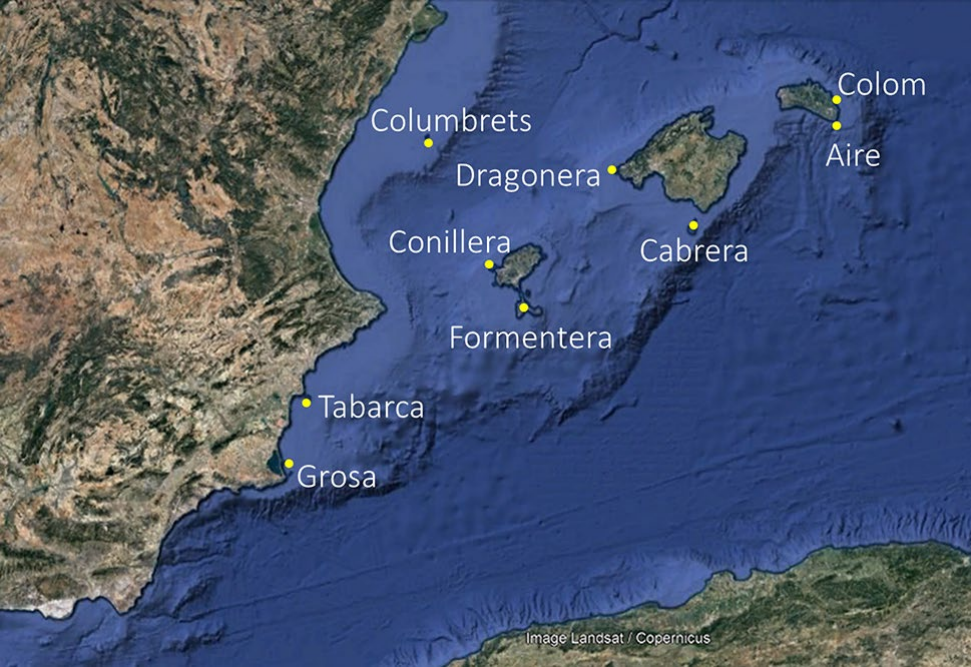
Geographical location of studied islands in the western Mediterranean. Image source: Google Earth. Data SIO, NOAA, US Navy, NGA, GEBCO. Image Landsat/Copernicus.

Systematic ringing in spring on Mediterranean islands has been promoted by the Piccole Isole project since 1988^26^. Standard methods of the project involve ringing between 16th April and 15th May attempting to include the peak of the spring passage of long-distance migrants. Ringing is performed from dawn to nightfall using a constant number of nets within ringing stations placed at stable sites located at representative habitats in each island (Supplementary Table S1). The use of tape-lures is not allowed. We have compiled ringing data for all the Spanish Mediterranean islands that have been applying this methodology, with the exception of Mallorca and Menorca where the ringing stations were located in wetlands and captured a large percentage of local birds (Fig. 2, Table 1). The nine study islands are spread along a south-west to north-east gradient and, with the exception of Columbrets, they are distributed in pairs of similar longitude but different latitudes (Fig. 2). Ringing stations have been operating over a variable number of years (5–27 years), with the maximum number of ringing stations operating at the same time occurring between 2003 and 2010. To include between-year variation on islands that started ringing campaigns more recently we used data from the years 2003–2018.

**Table 1:** Period of activity of the ringing stations located on each island between the years 1992 and 2018.

Island	Years																											Used
	92	93	94	95	96	97	98	99	00	01	02	03	04	05	06	07	08	09	10	11	12	13	14	15	16	17	18	
Colom																**	**	**	**	**								5
Aire		*	*	*	*	*	*	*	*	*	*	**	**	**	**	**	**	**	**	**	**	**	**	**	**	**	**	16
Dragonera													**	**	**	**	**											5
Cabrera	*	*	*	*	*	*	*	*	*	*	*	**	*	**	**	**	**	**	**	**	*							8
Conillera												**	**	**	**	**	*	**	**									7
Formentera												**	**	**	**	**		**	**									7
Columbrets			*	*	*	*	*	*	*	*	*	**	**	**	**	**	**	**	*					*	**	*	**	9
Tabarca																		**	**	**	**	**	**	**	**	**	**	10
Grosa																*		**	*	**	*	**	**	**	**	**	*	7
Total	1	2	3	3	3	3	3	3	3	3	3	5	5	6	6	7	5	8	6	5	2	3	3	3	4	3	4	

* or ** identify years when the ringing station was operating on each island. **Identify years that fulfill the conditions described in the text to be used in analyses while *are years before 2003 or years that did not fulfill those conditions. The column Used shows the number of years whose data were used in analyses for each island.

The ringing period within each spring also varied in most islands, owing to funding or logistic limitations; thus, to reduce the possible effects on migrant composition we only used data from the standard period of the Piccole Isole project and from years that included at least one week of ringing in the fortnight of each month within this interval. This procedure excluded the use of some years for several islands, and the final number of data years for islands ranged between 5 and 16 (Table 1).

We used only data for trans-Saharan nocturnal migrant passerines, which form the bulk of species ringed on Mediterranean islands during the standard period. The standard ringing period only covers the tail end of the short-distance migrants’ passage; thus, these species were excluded as their contribution to composition of migrants could vary mainly due to between-year variation in migration phenology. Diurnal migrants, like hirundinids and fringillids, also represent a small fraction of birds ringed and may use different cues to select stopover islands. In addition, some of these species nest in some of the islands studied and birds ringed could include breeding birds. To avoid the distorting effect of species that are captured accidentally in very small numbers, we considered only the species that were ringed in at least five separate years, or on five different islands, which limited the species considered to 35 (Supplementary Table S2). This led to the exclusion of just two species (*Ficedula semitorquata* with three individuals ringed in two islands and *Locustella luscinioides* with one individual ringed in Aire island). In addition, we only considered the number of ringed birds, since the proportion of recaptures varies among islands, likely reflecting variation in the duration of stopovers^21^, which could bias the comparison of the patterns of migrant species composition.

### Island descriptors

We obtained two groups of variables describing the characteristics of the study islands (Tables 2, 3): (1) Variables related to geographical location: latitude, longitude, straight distance and minimum distance to the North African coast, minimum distance to the closest large body of land (continent or large island) in any direction and to the closest large body of land situated in a southerly angle between SW and SE. (2) Variables related to the habitat characteristics of the islands: area, maximum altitude and Normalized Difference Vegetation Index (NDVI). We estimated NDVI from Landsat 8 Images taken during the standard ringing period in the years 2015 and 2016. Pixels containing shoreline were excluded and the average NDVI was calculated for the rest of the pixels.

**Table 2:** Variables describing the characteristics of the islands that included the ringing stations studied.

Variable	Description
*Geographical location*	
Latitude	Decimal degrees North
LongitudeKm	Distance in km from the westernmost island, Grosa Island
MinDistAfrica	Minimum distance to North African coast in any direction, km
StrDistAfrica	Straight distance, north–south direction, to North African coast, km
MinDistLand	Minimum distance to the closest large body of land situated in any direction, km
MinDSouthLand	Minimum distance to the closest large body of land situated between SW and SE, km
*Habitat characteristics*	
Area	Area of the island, km2
MaxAlt	Maximum altitude, m.a.s.l
NDVI	Normalized Difference Vegetation Index in spring

**Table 3:** Values of the island descriptors (see Table 2) and two measures of temporal variability of migrant composition in each island: average local contribution of each island to beta diversity (LCBD) and beta diversity for each island (BD_Ti_).

Island	Latitude	Longitude	LongKm	MinDistAfrica	StrDistAfrica	MinDistLand	MinDSouthLand	Area (km^2^)	AltMax (m)	NDVI	LCBD	BD_Ti_
Aire	39.80	4.29	431.2	321	321	1.1	321	0.30	7	0.239	0.008	0.038
Cabrera	39.14	2.95	318.4	258	258	13.7	258	11.53	175	0.274	0.027	0.086
Formentera	38.73	1.40	184.7	232	234	2.1	234	83.20	195	0.229	0.012	0.048
Colom	39.96	4.28	429.4	341	341	0.28	0.61	0.59	43	0.301	0.008	0.040
Columbrets	39.90	0.69	122.1	377	395	50	108	0.14	67	0.144	0.007	0.033
Conillera	38.99	1.22	168.4	270	273	1.2	1.2	0.72	59	0.216	0.014	0.059
Dragonera	39.58	2.32	262.8	312	326.5	0.8	2.75	2.52	353	0.311	0.029	0.154
Grosa	37.73	-0.71	0	193	219	2.4	9.5	0.18	97	0.153	0.009	0.024
Tabarca	38.17	-0.47	22.8	228	252	4.8	61.7	0.40	15	0.159	0.017	0.070

### Continental abundance data

Abundance estimates for western Europe were obtained from the European Red List of Birds^27^. We used the mean of the minimum and maximum number of pairs estimated for the 27 EU Member States as a measure of continental abundance (Supplementary Table S2).

### Data analysis

All analyses were done using R 3.6.1^28^. We built a matrix of island-year x species containing the number of individuals of each selected species ringed in the study period in each island and year. Average number of individuals of each species ringed at each island was calculated and was used (log-transformed) as a dependent variable in a linear model with continental abundance (log-transformed), island and their interaction as predictors. This model was simplified using AICc as criteria to identify the best model.

To analyze variation of species composition, the matrix of island-year x species was transformed using the chord transformation^29^ with the function decostand in the vegan package^30^.

Using the function beta.div of the adespatial package^31^ we calculated beta diversity, including temporal and between-island variability (BD_I,T_), as the total variance of the aforementioned transformed matrix (BD_Total_ in^29^), which varies between 0 and 1 when chord distance is used. Considering that y_ijk_ is the chord transformed abundance of the species j in the island i and year k and $\overline{{y }_{j}}$ is the mean for species j in all islands and years altogether, then:

$${SS}_{Total}=\sum_{i=1}^{n}\sum_{j=1}^{p}{\sum_{k=1}^{q}{({y}_{ijk}-{\overline{y} }_{j})}^{2}}$$

$$BD_{I,T} = \, SS_{Total} /\left( {N - 1} \right)$$

where N is the total number of samples. The function beta.div also provides an estimation of contribution of localities (LCBD) and species (SCBD) to beta diversity (Table 3). Yearly LCBD (log transformed because of skewed distribution) of each island were averaged and compared between islands using ANOVA and a post-hoc Tukey test.

We partitioned the above sum of squares in several ways. First, we calculated a beta diversity that considered only between-island variability, excluding temporal variability (BD_I_), by averaging the chord transformed abundances of each species *j* in each island along study years (${\overline{y} }_{ij}$) and applying the same procedure, but using the number of studied islands (n):

$${SS}_{I}=\sum_{i=1}^{n}\sum_{j=1}^{p}{{({\overline{y} }_{ij}-{\overline{y} }_{j})}^{2}}$$

$$BD_{I} = SS_{I} /\left( {n - 1} \right)$$

Second, we calculated a beta diversity due to inter-annual variation of migrant composition within islands (BD_T_) as:

$${SS}_{Temp}=\sum_{i=1}^{n}\sum_{j=1}^{p}{\sum_{k=1}^{q}{({y}_{ijk}-{\overline{y} }_{ij})}^{2}}$$

$$BD_{T} = SS_{Temp} /\left( {Y - n} \right)$$

where Y is the total number of study years and n is the number of studied islands (9). We also calculated a temporal beta diversity for each island *i* (BD_Ti_) as the sum of squares due to variation within the island divided by the number of the island study years (Yi) minus 1:

$${SS}_{Temp,i}=\sum_{j=1}^{p}\sum_{k=1}^{q}{({y}_{ijk}-{\overline{y} }_{ij})}^{2}$$

$$BD_{Ti} = SS_{Temp,i} /\left( {Y_{i} - 1} \right)$$

Differences in temporal variability between islands could be due to different predominance of species that are more or less variable between years. To check this, we calculated Spearman’s rank correlation between the percentage of captures of each species in the total ringed on each island and BD_Ti_ and LCDB indices, for species present on all islands.

We tested for the existence of differences between islands in migrant species composition using Permutational Multivariate Analysis of Variance (PERMANOVA) using the function adonis2 in the vegan package. We performed a multivariate test of homogeneity of variances using the betadisper function (vegan package) with the adjustment for small sample bias, to test if temporal variability in species composition differed between islands. We made post-hoc comparisons between islands with False Discovery Rate (FDR) correction using the function pairwise.perm.manova of the package RVAideMemoire^32^.

To identify gradients in migrant species composition and the island characteristics that were associated with them, we employed Redundancy Analysis using the rda function (vegan package). We used the chord transformed matrix of species x island-year as a response matrix. We used two explanatory matrices, one including variables of geographical location and the other the variables related to habitat characteristics of the islands. We evaluated the relative importance of each group of variables to explain migrant species composition by performing a variation partitioning analysis, using the varpart function (vegan package). For that analysis, we followed the steps and R scripts recommended in^33^.

Variables describing island characteristics were transformed using natural logarithms and collinearity within each group was evaluated with variance inflation factor (VIF)^34^. All the habitat variables presented VIF < 3 and were retained in subsequent analyses, but some of the geographical location variables presented VIF values much larger than 10, so we removed the variable with the highest VIF and recalculated VIF values. This procedure was repeated until all variables presented VIF < 10 and led to the exclusion of minimum distance to Africa and latitude. Maximum VIF in the remaining variables was 2.1. We then performed a separate forward selection of variables within each group using the vegan function ordistep and all variables were selected.

A second redundancy analysis was performed with all the explanatory variables together to identify the particular variables most related to gradients of composition of migrants. Because we have repeated measures for each island, we fitted linear mixed models to identify the explanatory variables significantly related to each RDA axis, using the function glmmTMB (glmmTMB R package^35^). In these models, scores of each RDA axis were dependent variables, each island descriptor was tested as an explanatory variable and the island identity was included as a random effect. To evaluate the species whose abundance was significantly related to RDA gradients, we calculated correlation coefficients of each species abundance (chord transformed) and RDA axis scores.

### Models of species response

To evaluate the response of each study species to the geographical and habitat characteristics of the islands, we calculated the average number of individuals captured daily per 100 m of mist net in each study year in each island. However, the capture index from Columbrets Island is not comparable to the other islands because most birds were trapped in just three nets placed around a clump of isolated *Opuntia* cactus, which strongly attracted birds landing on this island. On the other islands, nets were set in several straight lines across the available habitat, and thus sampling was more uniformly distributed. The relationship between the percentage of total captures and the number of birds captured per 100 m of net is similar in all islands except Columbrets (Supplementary Fig. S1 online) and thus it was not possible to include Columbrets in the models for the captures/100 m net.

To check if excluding Columbrets from these analyses changed the results, the species response to island variables was modeled twice: using as a dependent variable the proportion of captures, including Columbrets, and using an abundance index calculated as the average number of birds captured daily per 100 m of mist net (excluding Columbrets). In both cases, we used generalized linear mixed models (GLMM, R function glmmTMB). In the proportion of captures models, we included the natural logarithm of the total number of birds ringed of the selected species in each island and year as an offset. For the captures/100 m net models, the offset was calculated as the natural logarithm of the result of multiplying the total length of nets (in hundreds of meters) by the number of days that the nets were operating in each spring. Island identity and year, considered as a factor, were included in the models as random effects. Each of the island variables included in the RDA, after excluding colinear variables, was included in turn as a fixed effect in these GLMMs. We considered three potential family distributions: Poisson and two parameterizations of the Negative Binomial distribution (nbinom1: variance increases linearly with the mean; nbinom2: variance increases quadratically with the mean). A null model including only the random effects was run with each family distribution and the one with the lowest AICc was selected. Then, linear and quadratic models including by turn one of the island descriptors were fitted and retained if it had an AICc lower than the null model. For each species we considered as equally plausible the models with ΔAICc ≤ 2. We used the performance_aicc function from the performance package^36^ to calculate AICc. Significance of regression coefficients in quadratic models was checked by comparing quadratic and linear models with the anova function.

### Effect of wing shape on island selection

We used the Kipp index as an indicator of wing pointedness^37^. This index is calculated as the percentage of the distance from the tip of the first secondary feather to the tip of the longest primary feather (primary projection) of the wing length (Kipp = 100*primary projection/wing length)^38^. Primary projection was measured with a transparent rule placed over the folded wing and wing length was measured as the maximum length with a butted ruler^38^, both with a precision of 0.5 mm. Primary projection data are only available for Tabarca Island from 2018 onwards, so these measures were lacking for some of the species less frequently ringed in the study area. The primary projection of great reed warblers (*Acrocephalus arundinaceus*) and wood warblers (*Phylloscopus sibilatrix*) were obtained from a nearby ringing station in the mainland (Hondo Natural Park). The Kipp index of individuals were averaged to obtain mean and SD for each species. We also used the wing aspect ratio (WAR) available in^39^ for most of the species studied.

We tested if mean Kipp index and wing aspect ratio differed between groups of species whose abundance was related to different RDA axes or selected island variables using phylogenetic generalized least-squares (PGLS) models^40^ constructed using the R package ape^41^ and nlme^42^. These models allow to account for potential non-independence among species due to shared evolutionary history by estimating a phylogenetic signal index, Pagel’s λ^43^, that measures phylogenetic dependence of observed trait data. For that aim, following^44^ 2500 trees were downloaded from Birdtree web site^45^ for the set species with Kipp index available and the same quantity of trees for the species with WAR estimates available. A consensus phylogenetic tree was built for each set of species using the R package phytools^46^ (Supplementary Figs. S4 and S5 online). *Iduna opaca* was not available in Birdtree data and was substituted by the closely related *Hippolais pallida* (currently named *Iduna pallida*), from which it was split. Bivariate PGLS models including interaction were fitted and interaction was removed if not significant. Models were fitted with estimated Pagel’s λ and with λ fixed to zero (i.e. no phylogenetic signal). Likelihood ratio test was used to compare these models and if not significant the simplest model was selected^47^.

## Results

The total number of birds ringed of each selected species in the study years is shown in Supplementary Table S2. Half of the species (18 out of 35) were ringed on all islands and represent 98.6% of all ringed birds analyzed. The species most often ringed on all the islands was the willow warbler (*Phylloscopus trochilus*), representing between 30 and 61% of all birds ringed.

**Figure 3:**
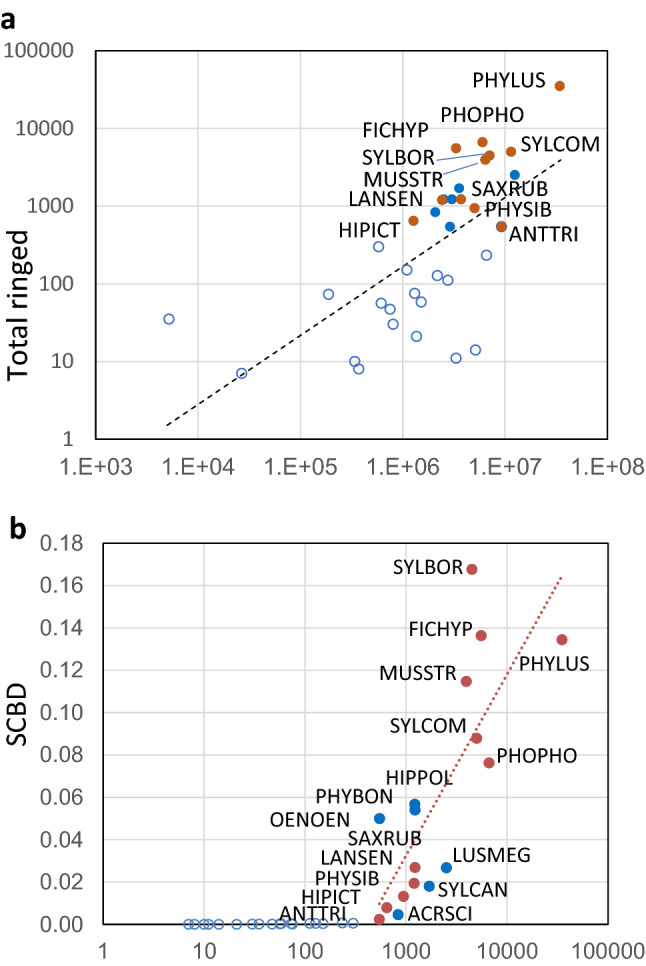
(**a**) Relationship between continental abundance (horizontal axis) and total number ringed (both in logarithmic scale). (**b**) Relationship between the total number ringed (horizontal axis, logarithmic scale) and their SCBD (Species Contribution to Beta Diversity). Hollow circles: species not captured on all islands. Filled circles represent species captured on all islands differentiating between species correlated (red circles) with RDA 1 (see Fig. 4) or not correlated with that axis (blue circles). Regression line in b fitted for species ringed on all islands only. Acronym identification in Supplementary Table S2. For clarity, only species acronyms for red circles are shown in a.

The model for the relationship of continental abundance and the number of birds ringed on each island that included an additive effect of island was better than the model with the interaction (ΔAICc = 12.04), but the model without an effect of island was even better (ΔAICc = 6.05). The model with only the island predictor and the null model were clearly worse (ΔAICc > 128 in both cases), thus this result supports the hypothesis that continental abundance has a positive significant effect on species abundance at stopover sites and that this effect is similar in all islands studied (Supplementary Fig. S4 online). To check if this could be an effect of the large continental abundance of the willow warbler, which is almost an order of magnitude greater than any other species and could be an influential point, models were fitted excluding this species but the result was the same. Therefore, a model relating logarithm of continental abundance of each species to logarithm of number ringed on all islands was finally fitted (Log(ringed) = 0.8875 × Log(Continental abundance) −3.1019; F_1,33_ = 26.03; p < 0.001; r = 0.664; Fig. 3a).

### Beta diversity

Total beta diversity was low (BD_I,T_ = 0.098). Beta diversity excluding temporal variability (BD_I_ = 0.056) was very similar to the average beta diversity within islands caused by temporal variability (BD_T_ = 0.056). The average contribution of islands to beta diversity (LCBD, Table 3) was different among islands (F_8,65_ = 3.49, p < 0.01), due mainly to significant differences between Cabrera and Columbrets and Aire (Tukey-post-hoc tests p < 0.01) and marginally significant differences between Tabarca and the same two islands (Tukey-post-hoc tests p < 0.1). BD_Ti_ and LCDB were highly correlated (r = 0.912, p < 0.001) showing that islands that were more variable interannually contributed more to beta diversity. The only island variable related to BD_Ti_ and LCDB was the maximum altitude of the island (BD_Ti_: r = 0.787; LCDB: r = 0.729, both p < 0.05). However, this result is due to the high variability of Dragonera, the highest island, and if Dragonera is dropped from the analysis, this correlation disappears. Neither BD_Ti_ nor mean LCBD were correlated with the number of years of study on each island (BD_Ti_: r = −0.361; LCDB; r = −0.339, n.s.).

Species Contribution to Beta Diversity (SCBD) was positively correlated to their ringed total (r = 0.613 p < 0.001). The main outlier of this trend was the willow warbler, by far the most ringed species, which had a SCBD lower than expected for its abundance (Table 4, Fig. 3b). Species ringed on all islands accumulated almost all SCBD (0.9966) showing that beta diversity was due to changes in abundance of these migrants between islands. The only species with a significant correlation with indices of temporal variability between islands was the willow warbler (LCDB; r_s_ = −0.700, p = 0.043; BD_Ti_: r_s_ = −0.817, p = 0.008; df = 7). After excluding the most variable island (Dragonera), the outcome is similar (LCDB; r_s_ = -0.690, p = 0.058; BD_Ti_: r_s_ = −0.857, p = 0.007; df = 6). Therefore, the beta diversity of islands where willow warbler represents a larger proportion of migrants tends to be less variable between years.

**Table 4:** Contribution of each species to beta diversity (SCBD) and correlation of their abundance index (chord transformed) with the three significant axes obtained from RDA.

Species	Number of islands	SCBD	RDA1 (24.2%)		RDA2 (8.7%)		RDA3 (6.0%)	
ACRARU	7	0.00011	-0.439	***	-0.037		0.391	***
ACRSCH	8	0.00038	**-0.604**	***	-0.383	***	0.413	***
ACRSCI	9	0.00456	0.014		-0.366	**	0.124	
ANTCAM	7	0.00011	-0.238	*	-0.168		0.174	
ANTTRI	9	0.00229	-0.412	***	0.036		0.376	***
CALBRA	5	0.00027	-0.250	*	-0.073		0.191	
CERGAL	6	0.00003	-0.025		-0.363	**	-0.069	
EMBHOR	7	0.00030	0.029		-0.332	**	0.180	
FICALB	5	0.00003	-0.421	***	0.012		0.103	
FICHYP	9	0.13628	**-0.764**	***	0.024		**-0.457**	***
HIPICT	9	0.00787	-0.399	***	-0.113		0.325	**
HIPPOL	9	0.05661	0.137		**-0.507**	***	-0.337	**
IDUOPA	6	0.00013	0.054		**-0.472**	***	-0.200	
LANCOL	4	0.00001	-0.090		0.086		0.146	
LANSEN	9	0.01943	-0.323	**	-0.231	*	-0.135	
LOCNAE	9	0.00053	0.122		0.094		-0.012	
LUSMEG	9	0.02676	0.213		**-0.513**	***	-0.120	
LUSSVE	5	0.00001	0.013		-0.149		-0.067	
MONSAX	3	0.00001	-0.150		-0.086		0.115	
MOTFLA	8	0.00062	-0.040		-0.192		0.256	*
MUSSTR	9	0.11475	**-0.496**	***	0.272	*	-0.212	
OENHIS	8	0.00006	0.082		-0.234	*	-0.121	
OENOEN	9	0.04993	0.128		**-0.725**	***	-0.207	
ORIORI	8	0.00037	-0.381	***	-0.106		0.199	
PHOPHO	9	0.07629	**-0.523**	***	**-0.643**	***	-0.159	
PHYBON	9	0.05404	-0.110		-0.016		**-0.828**	***
PHYIBE	8	0.00016	-0.025		0.004		**-0.455**	***
PHYLUS	9	0.13438	**0.892**	***	0.414	***	0.163	
PHYSIB	9	0.01317	**-0.698**	***	0.109		-0.018	
SAXRUB	9	0.02681	**-0.800**	***	-0.211		0.251	*
SYLBOR	9	0.16768	**-0.789**	***	-0.269	*	0.398	***
SYLCAN	9	0.01794	0.096		**-0.704**	***	-0.182	
SYLCOM	9	0.08781	**-0.643**	***	**-0.603**	***	0.227	
SYLCUR	3	0.00000	-0.005		0.145		0.077	
SYLHOR	8	0.00026	0.057		**-0.569**	***	-0.259	*

Parentheses in each axis heading show the percentage of total variability explained by each axis. Number of islands where each species was ringed in at least one year is also shown. Species acronyms identification in Supplementary Table S2. *p < 0.05, **p < 0.001, ***p < 0.001. Significant correlations after Bonferroni correction are shown in bold, and marginally significant correlations (p < 0.1) after that correction are underlined.

The PERMANOVA test yielded significant differences in migrant composition between islands (F_8,65_ = 7.89, p < 0.001) and the permutational post-hoc test detected significant differences between all islands except between Dragonera and Colom and only marginally significant differences between Aire and Columbrets. However, as the betadisper test for multivariate homogeneity of variance was significant (F_8,65_ = 2.53, p < 0.05, Tukey post-hoc test: only differences between Dragonera and Grosa and Aire p < 0.05), this result could be influenced by the heterogeneity of variances. This analysis was repeated excluding Dragonera and then the betadisper test was no longer significant (F_7,61_ = 1.59, p > 0.1) while PERMANOVA produced a very similar result (F_7,61_ = 8.88, p < 0.001). Post-hoc comparisons yielded the same result of migrant species composition being different between all islands with the exception of Aire and Columbrets, whose difference was marginally significant (p < 0.1).

### Gradients of migrant species composition

The partitioning variance analysis performed with all variables indicated that the island descriptors explained close to 40% of variance (R^2^_adj_ = 0.391) that was partitioned in R^2^_adj_ = 0.140 explained by geographical variables (ANOVA permutational test for rda: F_4,66_ = 5.02, p < 0.001), R^2^_adj_ = 0.127 explained by habitat variables (ANOVA permutational test for rda: F_3,66_ = 5.82, p < 0.001) and R^2^_adj_ = 0.124 (not testable) explained jointly by the two data sets. Geographical and habitat characteristics have weak correlations except for NDV and Longitude; therefore, the position of the study islands along the west–east direction appears to be the main link between habitat and geographical variables.

The RDA performed with all selected island variables together identified as significant the first three axes (p < 0.01 in all cases), which accounted for 86.5% of the variance explained by the model and 38.9% of total variance (Table 4). The first RDA axis (RDA1) was related to the island habitat variables and Longitude (Table 5). Axis 2 (RDA2) and 3 (RDA3) were related to distance to the closest large body of land situated in a southerly direction (MinDSouthLand), but this relationship was negative in the former and positive in the latter (Table 5). RDA2 was also negatively related to the distance to the large body of land in any direction (MinDistLand). None of the axes were related to the distance to the African coast.

**Table 5:** Correlation of island variables with significant RDA axis.

Variable	RDA1	p GLMM	RDA2	p GLMM	RDA3	p GLMM
**Geographical location**						
LongitudeKm	− 0.373	0.0403	0.184	ns	0.183	ns
StrDistAfrica	0.143	ns	0.297	ns	0.218	ns
MinDistLand	− 0.049	ns	− 0.420	0.0353	0.184	ns
MinDSouthLand	− 0.001	ns	− 0.355	0.0310	0.571	0.0042
**Habitat characteristics**						
Area	− 0.662	0.0000	− 0.090	ns	0.069	ns
MaxAlt	− 0.522	0.0113	0.147	ns	− 0.101	ns
NDVI	− 0.520	0.0032	0.365	ns	0.086	ns

The p values are obtained from univariate mixed models with island identity as a random effect. ns: non-significant.

**Figure 4:**
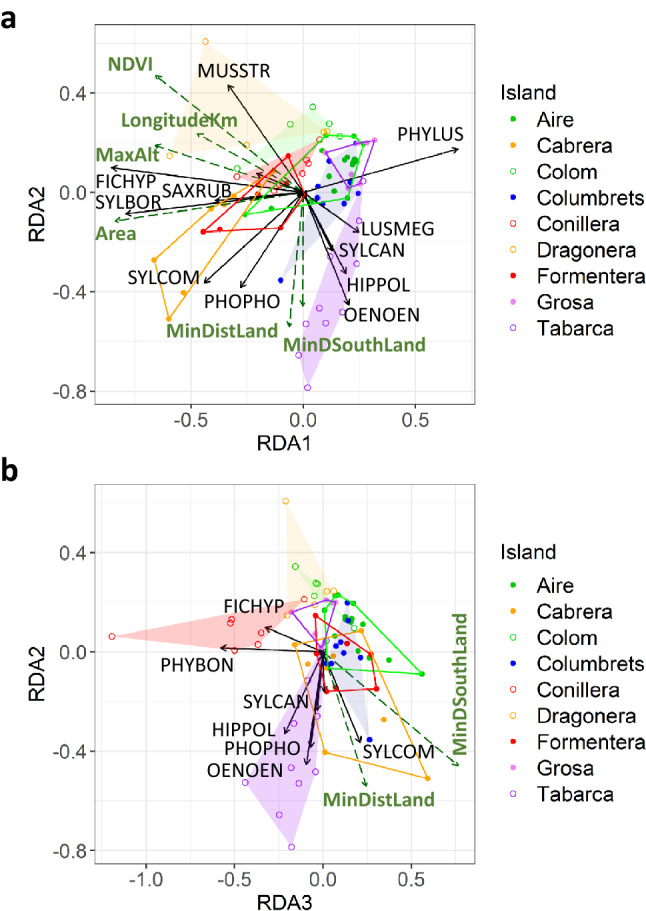
Triplot of the RDA relating migrant species composition to island variables. Each point corresponds to a spring migration year on one island. Pairs of islands located at similar longitude are depicted with the same color. Within each pair, points of the island with a more southern position are represented with a filled circle and hollow polygons and points of the island in the northern position with a hollow circle and shaded polygons. Dashed dark green arrows represent island variables significantly related to at least one of the axes represented (Table 5). Continuous black arrows represent the species most correlated to the axis (Table 4). The small unlabeled arrow in panel a corresponds to PHYSIB and in b corresponds to LUSMEG. Species acronyms identification in Supplementary Table S2. Figures were produced using R (R 3.6.1, https://www.R-project.org).

Yearly variation in migrant composition in each island tends to be distributed in a particular space in the resulting ordination (Fig. 4). Points of smaller islands are mostly located towards the right side of the first axis and those of the larger islands (Cabrera, Dragonera and Formentera) towards the left. Ordination in axis 2 and 3 separates islands that are very close to large bodies of land located south (Conillera, Dragonera and Colom) from islands that are far from the nearest coast in a southerly direction. The only species related positively to RDA1 was the willow warbler, while the rest of the species significantly related to this axis show a negative correlation (Table 4), with whinchat (*Saxicola rubetra*), garden warbler (*Sylvia borin*) and pied flycatcher (*Ficedula hypoleuca*) being the most important. The most abundant migrant species on those islands were correlated with RDA1 (Fig. 3b), thus its abundance seems related to habitat characteristics of islands. The most significant correlations of species with RDA2 were negative, supporting the idea that abundance of these species is greater on islands that are far from a southern coast.

### Response of species to island characteristics

The variable most often included in significant models (Supplementary Tables S3 and S4; Fig. S5 online) is the distance to the main area of land located south (Table 6), both for explaining the percentage of captures and the standardized number of birds ringed (abundance index). The position of the island in the east–west gradient is also important in both groups of models. Among the island characteristics, island area and NDVI are the variables most often included in both groups of models. Variables had a more consistent effect when used to model abundance index, thus in 20 of the studied species abundances increased with distance to southern land, in 10 species it increased with island area and in 9 with longitude. Distance to the northern coast of Africa was the least important variable.

**Table 6:** Number of species with a significant model relating the percentage of total captures or the average daily number of captures per 100 m of mist net to island descriptors.

Variable	Percentage of captures			Captures/100 m net		
	Linear	Quadratic	Total	Linear	Quadratic	Total
**Geographical**						
MinDSouthLand	8 (5/3)	8 (1/0)	16 (6/3)	12 (12/0)	11 (8/0)	23 (20/0)
LongitudeKm	6 (4/2)	8 (1/0)	14 (5/2)	10 (9/1)	2 (0/0)	12 (9/1)
MinDistLand	1 (1/0)	1 (0/0)	2 (1/0)	5 (5/0)	4 (0/0)	9 (5/0)
StrDistAfrica	0	3 (0/0)	3 (0/0)	2 (0/2)	3 (1/0)	5 (1/2)
**Island features**						
Area	9 (6/3)	5 (1/2)	14 (7/5)	10(10/0)	-	10 (10/0)
NDVI	13 (6/7)	1 (1/0)	14 (7/7)	3 (1/2)	7 (0/0)	10 (1/2)
MaxAlt	6 (1/5)	2 (1/0)	8 (2/5)	3 (0/3)	4 (1/0)	7 (1/3)

In the captures/100 m net models, Columbrets Island is not included. Linear models and models with a quadratic term were tested. In parentheses the number of models with a positive (left) or negative (right) effect of each variable. The difference between the sum of numbers in parentheses and the total is due to models in which the maximum of captures occurs at intermediate values of the predictor variable.

**Figure 5:**
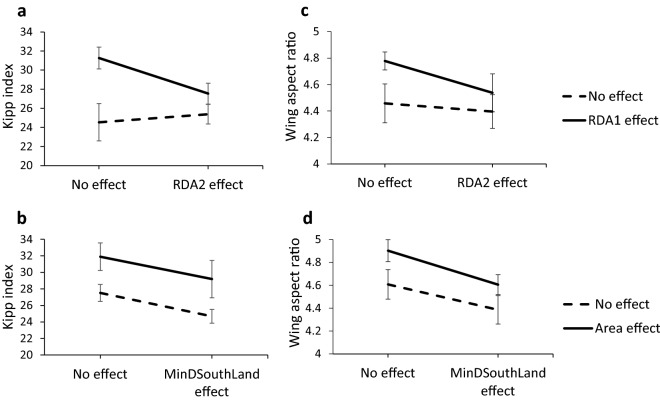
Mean (± SE) Kipp index (**a**) and wing span ratio (**c**) for species classified as presenting a significant negative correlation with RDA1 or RDA2 or not (No effect) (Table 4). Mean (± SE) Kipp index (**b**) and wing span ratio (**d**) for species classified as having a significant positive effect of the variables area or MinDSouthLand on its abundance index or not (No effect) (Table 3S). Minimum values in Y axis of both variables correspond approximately to the values for two short distance migrant species (*Erithacus rubecula* and *Phylloscopus collybita*).

There was a strong correlation (r = 0.885, p < 0.001) between the Kipp index for the study species ringed in Tabarca and wing aspect ratio compiled by^39^ from wild birds in Central Europe (Supplementary Table S5), which supports the idea that the Kipp index is a good surrogate for wing aspect ratio. PGLS models comparing mean Kipp index and wing aspect ratio of groups of species that were related to the main RDA axis or to the most important variables in species response models yielded very similar results. Interaction was not significant in any case and was removed from all the models. According to likelihood ratio test Pagel’s λ was retained in the model relating Kipp index to RDA axes (λ = 0.802) and the model relating WAR to Area and distance to the main area of land located south (λ = −0.645). Species whose abundance is negatively correlated with RDA1 had on average more pointed wings (Fig. 5) than species not correlated with this RDA axis (Kipp index: t_22_ = 2.78, p < 0.05; WAR: t_21_ = 2.22, p < 0.05), while there is no effect of correlation with RDA2 (Kipp index: t_22_ = 1.20, n.s.; WAR: t_21_ = 1.38, n.s.). When species were classified as related or not related to island area and to the distance to the main area of land located south there was a significant effect of island area (Kipp index: t_22_ = 3.34, p < 0.01; WAR: t_21_ = 3.70, p < 0.01) and MinDSouthLand (Kipp index: t_22_ = 2.17, p < 0.05: WAR: t_21_ = 4.11, p < 0.01). Species whose abundance responded to island area had more pointed wings while species related to distance to the land located south had more rounded wings (Fig. 5).

## Discussion

Most of the migrant species were present on all islands and summed to more than 98% of total birds ringed during the study years. The abundance index of passerine species at stopover sites was correlated with their continental abundance on all islands. Therefore, beta diversity is low and well below its possible maximum value of one. Despite this, when relative abundance of migrants is considered, virtually every island has its own pattern of migrant passerine composition, even islands that are located near each other. On the contrary, the most similar islands in terms of migrant composition (Aire and Columbrets) are situated almost at the extremes of the longitudinal gradient studied. Therefore, distance between islands at the scale of this study is not a determinant for their differences in migrant composition and other causes should be involved.

About half of the beta diversity is explained by inter-annual variation within islands. The difference between islands in temporal variability is not an artifact due to their different lengths of monitoring periods. Cabrera and Dragonera were the most variable islands, even though they were studied for 8 and 5 years respectively, while Aire is one of the least variable islands despite having been monitored for the longest period of the study set (16 years). Differences in temporal variability of migrant composition between islands could be explained by geographical or habitat characteristics, but we did not detect consistent effects of any of these variables. An alternative is that differences in temporal variability originate from differences in species composition. Given that continental abundance determines to a large extent migrant abundance, if populations of some species are more variable interannually^48–50^ and these species are more abundant on some islands, those islands would present more variation in migrant composition between years. In addition, some species could be more sensitive than others to variation in meteorological conditions^51,52^ which could cause more interannual variability in migrant composition. However, these potential effects are not detected because we did not find positive correlations between any species and island temporal variability indices, but only a consistent negative correlation with willow warbler relative abundance. Therefore, islands where willow warbler represents a larger proportion of migrants tend to be less variable between years. Given that among the species analyzed in this study the willow warbler is, by far, the most abundant in Europe and at the stopover islands, its high abundance might buffer between-year variability.

The variance partition analysis showed that geographical location and habitat characteristic variables explained one third each of the variation in migrant composition on the study islands, while the remaining third was explained conjointly by both sets of variables. Thus, the two types of variables are not completely independent, since some habitat characteristics change consistently with geographic position. This is due to the correlation between longitude and NDVI, which in this set of islands increases eastwards according to an increasing rainfall gradient from southwest to northeast.

The main gradient in species composition is strongly related to all the habitat variables and to a lesser extent to the longitudinal position of the island. The other two important RDA axes are only related to geographical variables, the distance to large pieces of land in any direction (MinDistLand) and in a southerly direction (MinDSouthLand). Willow warbler is the only species correlated to the gradient of habitat characteristics (RDA1) whose abundance appears to increase on small and dry islands. This is particularly striking since the remaining 16 species that present significant correlations with this axis are positively associated with island area. From Supplementary Table S2, it can be calculated that these 16 species together represent a similar number of birds (on average 43% of birds ringed on the study islands; range 24–64%) to the number of willow warblers ringed. Given that the chord transformed abundance is, like the percentage, a relative abundance index, when the abundance of the group of species positively related to larger islands increases, the relative abundance of willow warblers should decrease and in fact their chord transformed abundances are negatively correlated (r = −0.897).

To adequately interpret these results, it is necessary to analyze the models for species response to island variables. Response models for the species correlated with the habitat characteristics gradient are in general consistently positively related to area, NDVI or maximum altitude, regardless of whether the percentage of captures or the number of captures per 100 m of net is used as the response variable. On the contrary, in the case of the willow warbler the model for the percentage of captures is negatively related to area but the number of captures is independent of this variable. Therefore, the apparent preference of willow warbler for smaller islands is an artifactual consequence of about one-third of the species studied stopping in larger numbers on larger islands, thus decreasing the relative abundance of willow warblers on larger islands. Those species appear to be more selective of island habitat characteristics while willow warblers would stop in large numbers on any island available. It has been suggested that birds in poorer condition would be less selective of island characteristics and land at the first available site^21^. According to this idea, individuals of species whose abundance is related to island characteristics are expected to be on average in better condition. But contrary to expected, the average fat score of the species related to island characteristics is lower both in Columbrets (mean 1.2, SD 0.57, range: 0.6–2.5) and the Dry Balearic Islands (a group that includes all the Balearic islands analyzed here; mean: 2.0, SD: 0.37, range: 1.3–2.5) than the fat score of willow warbler (Columbrets mean: 1.8; Dry Balearic Islands mean: 3.0; data in^21^). The lower fat load of these species suggests that they would be on average in greater need of refueling and their relation to island habitat variables supports that they are more selective of stopover sites, in accordance with^5^. Refueling rates are often negative on dry small islands^21^ and thus it would pay, at least for individuals that are not in a desperate situation, to continue migration to a better stopover site. Given that small islands may provide other benefits apart from refueling^53,54^, the larger on average fat load of the willow warbler could explain their independence of island characteristics and weak relationship to island location.

The habitat variable that presents the most frequent and consistent positive effect in species response models for standardized numbers of captures was island area. Species models with NDVI and maximum altitude often included quadratic terms and predicted maximum or minimum numbers of captures at intermediate values of these variables, making their effect more unclear than with island area. This result supports the idea that island area may be an important clue used by migrants to assess island quality as a stopover site, as it may be the easiest characteristic to evaluate from afar. Alternatively, larger islands could be simply easier to detect from greater distances and would attract a larger number of birds. However, this explanation seems unlikely in our study area because all the smaller islands, apart from Columbrets, are very close to larger islands or the mainland coast, which could attract migrants.

Species whose abundance is correlated to the other two RDA axes appear to be related to island geographical location. Species response models for the number of captures reveal that the distance to the closest large body of land situated in a broad southerly direction is significant and has a positive effect for most species (20 of the 35 species analyzed). Therefore, in most species, the farther the island from a large piece of land located broadly south, the larger the abundance of landed migrants, which is likely related to a greater proportion of individuals in need of resting. Conversely, this suggests that islands that are closely located northwards to a larger body of land (Conillera, Dragonera and Colom) tend to be overflown by a larger proportion of migrants that made stopovers on the land that appeared just before. This is easily seen in the graphics for species response models since the above-mentioned islands have a counterpart located south of the same large Balearic island (Formentera, Cabrera and Aire) where the abundance of these migrants is considerably greater, despite being practically at the same longitude and in some cases very close by (see Aire and Colom). This result agrees with the prediction of optimal migration theory for the time minimizing strategy, which predicts that fuel gain at the best sites would allow skipping less optimal sites^5^.

On the other hand, some of the islands are very close to the Iberian Peninsula or a large Balearic island located to the north or northwest (Grosa, Tabarca, Formentera and Aire). Thus, birds could easily overfly these islands to arrive at the larger land. This does not seem to occur because in general birds arrive in large numbers to these islands even though trophic resources would be more abundant in the mainland. This also occurs in the Tyrrhenian Islands and has been explained by the idea that recovery after a long flight would first require rest^54^. Stopover at those islands tends to be in any case very short^21,54^.

The pattern that emerges from our results is that variation in migrant composition is determined mainly by two variables, island area and island distance to large pieces of land located in a broad southerly direction. Island area affects about a third of studied species that are more abundant at larger islands. Furthermore, abundance of most migrant species increases the farther an island is from other land that they could have encountered previously. Different species appear to be responding in different ways to these two variables and the analyses of primary projections and wing aspect ratios reported here support that wing morphology contributes to defining species' responses to island characteristics and geographical location. This supports the proposal of a hypothesis on the mechanism that may generate the differences in passerine migrant composition between islands.

Wing pointedness is related to the efficiency of long-distance flight and pointed wingtips have been shown to be one of the factors that reduce flight costs for small birds during migration^55,56^. Moreover, mass-adjusted wing aspect ratio has been found to be positively related to migration distance^57^. This suggests that individuals of species with more pointed wings crossing the Mediterranean would be more able to make longer flights, which would give them a better capacity to skip low quality islands and select better stopover islands, if they appear, or fly until the continent. Landing at more extensive islands may be advantageous as these islands have a more developed vegetation and a larger diversity of habitats, that would allow migrants to select the best habitat and reach a higher fuel deposition rate. On the contrary, small Mediterranean islands offer a very limited variety of dry scrublands, where fuel deposition rates have been found to be lower than in large Balearic Islands^21^. Migrating passerines arriving at unfamiliar stopover areas must base their choices on instant decisions^58^ and island area may be a quick and easy indicator to use. Low-quality stopover sites can act as an ecological trap, especially for migrating birds with insufficient fuel load to leave in search for an alternative place of a higher quality^58^. Moving to an alternative stopover site may be even more costly and unpredictable when crossing the sea, thus the use of cues for improving the chance of selecting a high-quality island or avoiding low quality ones would be highly rewarding.

On the other hand, it is revealing that species whose abundance is positively related to distance to land toward the south have on average a less pointed wing than species that do not present that relationship. Therefore, the higher flight costs expected for these species would determine that a relatively larger proportion of individuals need to land on the first available island, a proportion that would increase with the distance that birds had flown previously. The capacity of these species to select stopping islands according to their characteristics would be reduced, in comparison to species with more pointed wings, and their choice of stopover island may be more opportunistic. According to this hypothesis, larger islands would be enriched in individuals of migrant species with more pointed wings while on islands farther from southern land migrants with more rounded wings would reach higher abundance. Nevertheless, despite the importance of wing shape, it would be worth investigating which other morphological, physiological or ecological characteristics determine variation in stopover strategies and how they interact with weather variation. The importance of these drivers of migrant composition may be spatially dependent and thus could be different on sets of islands with different spatial distribution, such as the Tyrrhenian Islands, spreading along a north–south gradient along the Italian coast^24^. Understanding how drivers of migrant composition change on other sets of islands would contribute to a more general view of factors determining landing decisions.

## Supplementary Information

Supplementary Information.

## Data Availability

The datasets generated during and/or analyzed during the current study are available from the corresponding author upon reasonable request.
