# Supplementary material for: Drivers of migrant passerine composition at stopover islands in the western Mediterranean
Source: Sci Rep. 2022 Feb 21;12:2943. doi: 10.1038/s41598-022-06912-2 (PMC8861067; doi:10.1038/s41598-022-06912-2)
Supplement: Supplementary file 1 — Supplementary Information. [file 41598_2022_6912_MOESM1_ESM.pdf]

Supplementary Information

Title: Drivers of migrant passerine composition at stopover islands in the western Mediterranean.

Germán M. López-Iborra[1*], Antonio Bañuls[2], Joan Castany[3], Raül Escandell[4], Ángel Sallent[5] and Manuel Suárez[6]

* Author for correspondence. ORCID: 0000-0003-3045-5498

german.lopez@ua.es

[1] Departamento de Ecología/IMEM Ramon Margalef. Universidad de Alicante. Spain

[2] Grupo Local SEO-Alicante. SEO/BirdLife. Alicante. Spain

[3] Grup Au d'Ornitologia. Castelló. Spain

[4] Societat Ornitològica de Menorca, Ap. de correus 83, 07720 Es Castell. Spain

[5] Asociación de Naturalistas del Sureste. Murcia. Spain

[6] Grup Balear d'Ornitologia i Defensa de la Naturalesa (GOB). Palma de Mallorca. Spain

Table S1. Sampling effort, habitats and dominant plant species on the studied islands. Net length: total length of nets used on each island. Net length remained constant throughout the study years, with minor variations in Colom (range 193-205 m) and Dragonera (120 m in 2004 and 144 m in the other years). On these two islands average length is shown. Net groups: number of groups of nets.

Island	Net length (m)	Net groups	Habitats	Dominant species
Aire	168	10	Mediterranean scrubland with tamarix	Tamarix africana<br>Pistacia lentiscus<br>Olea europaea var. sylvestris
Cabrera	120	3	Mediterranean scrubland with Aleppo pines	Pistacia lentiscus<br>Juniperus phoenicea<br>Juniperus oxycedrus<br>Rosmarinus officinalis
Formentera	144	4	Mediterranean scrubland with Aleppo pines	Juniperus phoenicea<br>Asparagus horridus<br>Pinus halepensis<br>Cistus clusii
Colom	200	9	Mediterranean scrubland with Aleppo pines	Tamarix africana<br>Pinus halepensis<br>Olea europaea var. sylvestris<br>Phillyrea media<br>Pistacia lentiscus
Columbrets	36	2	Mediterranean scrubland	Suaeda vera<br>Lycium intrincatum<br>Withania frutescens
Conillera	204	15	Mediterranean scrubland	Juniperus phoenicea<br>Pistacia lentiscus<br>Rosmarinus officinali
Dragonera	140	4	Mediterranean scrubland with Aleppo pine	Pistacea lentiscus<br>Olea europaea var. sylvestris<br>Pinus halepensis
Grosa	132	5	Mediterranean scrubland	Lycium intrincatum<br>Whitania frutescens<br>Salsola oppositifolia
Tabarca	108	3	Mediterranean scrubland	Whitania frutescens<br>Lycium intrincatum<br>Opuntia maxima

Table S2. Number of individuals of each species ringed on each island in the selected study years. Six letter acronyms according to Busse and Kania (1989) (The Ring. 138-139: 169-178). Retraps are not included. Eur. Pop.: European population estimated for the 27 EU members according to BirdLife International.

Species	Acronym	Aire	Cabrera	Formentera	Colom	Columbets	Conillera	Dragonera	Grosa	Tabarca	Total	Eur. Pop.
Acrocephalus arundinaceus	ACRARU	36	20	5	5	2	1	6	0	0	75	1,310,000
Acrocephalus schoenobaenus	ACRSCH	52	45	8	4	11	2	2	0	3	127	2,180,000
Acrocephalus scirpaceus	ACRSCI	465	72	25	10	166	32	5	9	50	834	2,075,000
Anthus campestris	ANTCAM	17	4	16	1	5	0	0	1	3	47	753,000
Anthus trivialis	ANTTRI	217	83	71	7	103	14	31	12	6	544	9,245,000
Calandrella brachydactyla	CALBRA	14	2	28	0	13	1	0	0	0	58	1,515,000
Cercotrichas galactotes	CERGAL	2	0	1	0	1	1	1	0	2	8	372,500
Emberiza hortulana	EMBHOR	62	12	10	0	46	0	3	7	11	151	1,097,500
Ficedula albicollis	FICALB	8	8	1	0	0	2	2	0	0	21	1,361,500
Ficedula hypoleuca	FICHYP	1546	1247	522	352	409	743	355	188	192	5554	3,315,000
Hippolais icterina	HIPICT	398	101	32	44	32	18	8	3	9	645	1,275,000
Hippolais polyglotta	HIPPOL	243	35	94	10	238	172	24	116	289	1221	3,025,000
Iduna opaca	IDUOPA	12	6	0	0	0	3	1	2	11	35	5,200
Lanius collurio	LANCOL	10	1	0	0	1	0	2	0	0	14	5,145,000
Lanius senator	LANSEN	139	99	239	16	384	165	70	18	71	1201	2,420,000
Locustella naevia	LOCNAE	173	15	15	4	43	19	9	9	12	299	581,500
Luscinia megarhynchos	LUSMEG	1124	105	64	41	680	156	35	120	184	2509	12,480,000
Luscinia svecica	LUSSVE	3	1	0	0	3	1	0	0	2	10	340,500
Monticola saxatilis	MONSAX	1	0	3	0	3	0	0	0	0	7	26,750
Motacilla flava	MOTFLA	125	11	7	0	63	1	10	8	8	233	6,600,000
Muscicapa striata	MUSSTR	1256	503	267	240	627	338	412	145	150	3938	6,420,000
Oenanthe hispanica	OENHIS	5	2	2	0	6	1	1	8	5	30	805,000
Oenanthe oenanthe	OENOEN	77	14	26	1	66	7	5	35	314	545	2,890,000
Oriolus oriolus	ORIORI	27	16	28	5	22	7	4	0	2	111	2,770,000
Phoenicurus phoenicurus	PHOPHO	2379	1119	280	230	1057	505	296	277	491	6634	6,030,000
Phylloscopus bonelli	PHYBON	76	82	62	29	172	562	54	84	103	1224	2,510,000
Phylloscopus ibericus	PHYIBE	14	8	0	7	4	7	1	9	6	56	615,000
Phylloscopus trochilus	PHYLUS	15901	2844	1770	1351	5540	2263	1546	2135	1696	35046	34,300,000
Phylloscopus sibilatrix	PHYSIB	287	239	90	87	67	78	73	8	12	941	5,035,000
Saxicola rubetra	SAXRUB	273	502	161	8	68	32	120	35	24	1223	3,710,000
Sylvia borin	SYLBOR	1499	1202	356	179	650	258	187	67	87	4485	7,065,000
Sylvia cantillans	SYLCAN	574	158	54	37	417	104	39	103	207	1693	3,565,000
Sylvia communis	SYLCOM	1697	895	606	106	812	280	191	100	302	4989	11,450,000
Sylvia curruca	SYLCUR	9	1	0	1	0	0	0	0	0	11	3,315,000
Sylvia hortensis	SYLHOR	20	3	6	0	17	7	2	1	17	73	188,000
Total		28741	9455	4849	2775	11728	5780	3495	3500	4269	74592	

Table S3. GLMM models fitted to the proportion of birds ringed of each species in the total ringed in each island and year. Each model includes the natural logarithm of one island variable as a fixed effect and island identification and year as random effects (random intercepts). Null model includes only the random effects. Fam. refers to the family distribution selected in the best null model: P: Poisson; NB1: negative binomial parametrization 1; NB2: negative binomial parametrization 2. ΔAICc is the difference between each model AICc and the best model AICc. Only models that improve the AICc of the null model are shown. The sign of the model coefficients is included for each island variable. When two signs are shown, the model is quadratic and the first refers to the linear term and second to the quadratic term. Significance of the model coefficients is shown as p. When the model is quadratic, p refers to the significance of an ANOVA comparing quadratic and linear models. Best model for each species is shown in underlined bold font. Equally plausible models (ΔAICc ≤ 2) are underlined. Gray shaded areas identify models that are consistent whether proportion or number of captures is used as a dependent variable (see Table S3).

		Null Model		Best Model	Area			NDVI			MaxAlt			LongitudeKm			MinDistLand			MinDSouthLand			StrDistAfrica		
Species	Fam.	AICc	ΔAICc	AICc	ΔAICc		p	ΔAICc		p	ΔAICc		p	ΔAICc		p	ΔAICc		p	ΔAICc		p	ΔAICc		p
ACRARU	NB1	181.2	13.9	167.3	12.3	+	0.039	2.2	+	0.000	13.1	-/+	0.028	0.0	+/-	0.017				12.8	-/+	0.018			
ACRSCH	NB1	232.1	6.2	225.9	5.8	+	0.075	6.1	+	0.091				2.2	+	0.020				**<u>0.0</u>**	**-/+**	**<u>0.014</u>**			
ACRSCI	NB1	438.2	4.5	433.7							<u>0.8</u>	-	<u>0.004</u>				4.1	+	0.088	**<u>0.0</u>**	**+**	**<u>0.002</u>**			
ANTCAM	P	155.7	3.2	152.5	2.1	+	0.020													**<u>0.0</u>**	**+**	**<u>0.028</u>**			
ANTTRI	NB2	403.2	1.2	402.0	<u>0.2</u>	+	<u>0.050</u>				**<u>0.0</u>**	**-/+**	**<u>0.056</u>**							<u>0.5</u>	+	<u>0.054</u>			
CALBRA	NB2	150.4	2.4	148.0																**<u>0.0</u>**	**+**	**<u>0.042</u>**			
CERGAL	P	55.8	0.0																						
EMBHOR	NB1	252.2	14.7	237.5				12.7	-	0.020							11.6	+	0.025	**<u>0.0</u>**	**+/-**	**<u>0.001</u>**			
FICALB	P	96.7	10.8	85.8	5.1	+/-	0.009	3.6	+	0.003				**<u>0.0</u>**	**+/-**	**<u>0.001</u>**							7.7	+/-	0.006
FICHYP	NB2	708.3	8.7	699.6	<u>0.3</u>	+/-	<u>0.005</u>	**<u>0.0</u>**	**+**	**<u>0.000</u>**	8.1	+	0.066	7.4	+	0.038				7.8	-/+	0.042			
HIPICT	NB2	381.1	16.6	364.5				12.1	+	0.002				4.9	-/+	0.042				**<u>0.0</u>**	**-/+**	**<u>0.000</u>**	16.3	+/-	0.039
HIPPOL	NB2	503.1	14.1	489.0				5.2	-	0.000				**<u>0.0</u>**	**+/-**	**<u>0.000</u>**				13.4	+/-	0.021			
IDUOPA	P	133.0	6.5	126.6													2.3	+/-	0.003				**<u>0.0</u>**	**<u>+/-</u>**	**<u>0.004</u>**
LANCOL	P	73.8	3.4	70.4										**<u>0.0</u>**	**+**	**<u>0.059</u>**									
LANSEN	NB2	504.2	2.5	501.7										**<u>0.0</u>**	**+/-**	**<u>0.008</u>**									
LOCNAE	NB2	326.3	3.8	322.5							**<u>0.0</u>**	**-**	**<u>0.000</u>**												
LUSMEG	NB2	569.7	10.2	559.5	**<u>0.0</u>**	**-/+**	**<u>0.014</u>**	2.1	-	0.000	6.5	-	0.004												
LUSSVE	P	62.3	0.0																						
MONSAX	P	51.3	0.3	50.9										0.0	+/-	0.029				0.5	+	0.339			
MOTFLA	NB1	297.5	3.7	293.9	<u>0.7</u>	-/+	<u>0.031</u>	2.4	-	0.039							2.8	+	0.084	**<u>0.0</u>**	**+/-**	**<u>0.034</u>**			
MUSSTR	NB1	672.3	6.1	666.2				**<u>0.0</u>**	**<u>+/+</u>**	**<u>0.029</u>**	4.8	+	0.035	5.4	+	0.082	5.5	-/+	0.077	4.2	-	0.017			
OENHIS	P	132.2	11.9	120.4				7.2	-	0.003				**<u>0.0</u>**	**-**	**<u>0.000</u>**							11.7	-	0.096

OENOEN	NB2	395.0	6.9	388.1				1.7	-	0.001				**0.0**	**+/-**	**0.005**	5.8	+/-	0.050	0.5	+/-	0.003
ORIORI	NB1	231.9	7.5	224.5	**0.0**	**+/+**	**0.027**							2.3	+/-	0.046						
PHOPHO	NB1	667.6	0.0																			
PHYBON	NB2	497.6	2.9	494.6							1.8	+/-	0.060	2.3	-	0.242				**0.0**	**-**	**0.032**
PHYIBE	NB1	182.4	4.1	178.3										3.4	-	0.008				**0.0**	**-**	**0.001**
PHYLUS	NB1	880.9	4.6	876.3	**0.0**	**-**	**0.001**															
PHYSIB	NB1	482.1	10.6	471.6	7.8	+/-	0.083	**0.0**	**+**	**0.000**				3.5	+	0.001				7.5	-/+	0.007
SAXRUB	NB2	491.2	7.0	484.1	1.7	+	0.000				**0.0**	**-/+**	**0.006**									
SYLBOR	NB2	654.2	4.3	649.9	**0.0**	**+**	**0.002**	3.2	+	0.043				1.6	+	0.012				2.7	+	0.030
SYLCAN	NB2	554.1	7.7	546.4	4.3	-	0.005	**0.0**	**-**	**0.000**	6.5	-	0.042	7.2	-	0.073	7.1	+	0.065			
SYLCOM	NB1	663.1	4.6	658.5	**0.0**	**+**	**0.001**							4.6	+/-	0.097				0.3	+	0.002
SYLCUR	P	57.3	9.0	48.3				8.6	+	0.191	6.4	-	0.022	**0.0**	**+**	**0.083**				2.4	-/+	0.088
SYLHOR	P	191.9	10.5	181.4	9.0	-	0.029							**0.0**	**+/-**	**0.000**						

Table S4. GLMM models fitted to the number of captures of each species per 100 m of net in each island and year. Each model includes the natural logarithm of one island variable as a fixed effect and island identification and year as random effects (random intercepts). Null model includes only the random effects. Fam. refers to the family distribution selected in the best null model: P: Poisson; NB1: negative binomial parametrization 1; NB2: negative binomial parametrization 2. ΔAICc is the difference between each model AICc and the best model AICc. Only models that improve the AICc of the null model are shown. The sign of the model coefficients is included for each island variable. When two signs are shown, the model is quadratic and the first refers to the linear term and second to the quadratic term. Significance of the model coefficients is shown as p. When the model is quadratic, p refers to the significance of an ANOVA comparing quadratic and linear models. Best model for each species is shown in underlined bold font. Equally plausible models (ΔAICc ≤ 2) are underlined. Gray shaded areas identify models that are consistent whether proportion or number of captures is used as a dependent variable (see Table S3).

	Null Model			Best Model	Area			NDVI			MaxAlt			LongitudeKm			MinDistLand			MinDSouthLand			StrDistAfrica		
Species	Fam.	AICc	ΔAICc	AICc	ΔAICc		p	ΔAICc		p	ΔAICc		p	ΔAICc		p	ΔAICc		p	ΔAICc		p	ΔAICc		p
ACRARU	NB2	178.2	14.7	163.5				**0.0**	**-/-**	**0.001**				4.3	+	0.010				13.5	-/+	0.036			
ACRSCH	NB1	209.4	9.1	200.3										6.4	+	0.041				**0.0**	**-/+**	**0.014**			
ACRSCI	NB1	381.9	6.0	375.9							4.9	-	0.039				5.9	+	0.098	**0.0**	**+**	**0.000**			
ANTCAM	P	134.0	8.7	125.3	7.0	+	0.385													**0.0**	**+**	**0.001**			
ANTTRI	NB2	355.9	2.4	353.5	1.2	+	0.039				0.4	-/+	0.014							**0.0**	**+**	**0.009**			
CALBRA	NB2	119.8	5.5	114.4	3.9	+	0.045	**0.0**	**-/-**	**0.001**										2.2	+	0.016			
CERGAL	P	50.6	0.0																						
EMBHOR	NB1	216.1	10.7	205.4													9.4	+	0.072	**0.0**	**+/-**	**0.040**			
FICALB	P	94.8	5.0	89.8				0.8	-/-	0.020				**0.0**	**+/-**	**0.036**									
FICHYP	NB2	645.9	4.2	641.7	3.2	+	0.044	**0.0**	**-/-**	**0.048**				1.4	+	0.008									
HIPICT	NB2	352.5	14.9	337.6				11.9	-/-	0.031				9.7	+	0.001				**0.0**	**-/+**	**0.000**			
HIPPOL	NB2	442.5	10.4	432.1				**0.0**	**-**	**0.000**				6.7	+/-	0.026	4.1	+/-	0.006	9.4	+/-	0.032	7.2	-	0.006
IDUOPA	P	130.0	3.0	127.0													0.2	+	0.021	2.7	+	0.093	**0.0**	**+/-**	**0.006**
LANCOL	NB1	70.6	3.5	67.1										**0.0**	**+**	**0.037**							1.6	+	0.065
LANSEN	NB2	439.3	5.9	433.4	**0.0**	**+**	**0.000**																4.9	+/-	0.030
LOCNAE	P	285.0	2.8	282.3							**0.0**	**-/+**	**0.030**							**0.0**	**+**	**0.009**			
LUSMEG	NB2	495.5	3.4	492.1							**0.0**	**-**	**0.003**							2.6	+	0.050			
LUSSVE	P	51.0	0.0																						
MONSAX	P	34.5	2.6	31.9	**0.0**	**+**	**0.014**										1.3	+/-	0.015	1.7	+	0.441			
MOTFLA	NB2	246.2	4.9	241.3																**0.0**	**+**	**0.002**			
MUSSTR	NB1	592.5	4.7	587.8				**0.0**	**+**	**0.001**	2.4	-/+	0.012	2.3	+	0.013									
OENHIS	P	107.8	7.9	100.0										**0.0**	**-**	**0.000**	3.6	+/-	0.012				3.5	-	0.002

OENOEN	NB2	340.1	9.1	331.0				4.7	-	0.002				7.8	+/-	0.059	4.6	+/-	0.022	**0.0**	**+/-**	**0.002**	8.1	-	0.051
ORIORI	NB1	203.5	6.1	197.4	**0.0**	**+**	**0.001**	1.4	-/-	0.010				1.2	+	0.021				2.9	-/+	0.021			
PHOPHO	NB2	640.7	2.2	638.5													0.3	+	0.020	**0.0**	**+**	**0.013**			
PHYBON	NB1	442.9	0.0																						
PHYIBE	NB1	161.8	0.6	161.2	**0.0**	**-**	**0.130**																		
PHYLUS	NB2	864.4	1.3	863.1							1.1	-/+	0.043							**0.0**	**+**	**0.032**			
PHYSIB	NB1	429.1	11.3	417.8	10.0	+	0.039	**0.0**	**-/-**	**0.008**				4.4	+	0.000				9.6	-/+	0.013			
SAXRUB	NB2	449.4	2.8	446.7	1.1	+	0.024				0.7	-/+	0.026				1.4	+	0.030	**0.0**	**+**	**0.008**			
SYLBOR	NB2	595.3	3.0	592.4	2.1	+	0.048	2.8	-/-	0.089				2.4	+	0.061				**0.0**	**-/+**	**0.072**			
SYLCAN	NB1	482.9	2.8	480.0							2.1	-	0.053				0.8	+	0.021	**0.0**	**+**	**0.006**			
SYLCOM	NB1	609.2	10.3	598.8	9.2	+	0.038										8.6	+	0.021	**0.0**	**+**	**0.000**			
SYLCUR	P	59.6	7.4	52.2							5.3	-	0.009	**0.0**	**+**	**0.002**							6.8	+	0.096
SYLHOR	P	159.7	1.0	158.7																**0.0**	**+**	**0.072**	0.1	+/-	0.024

Table S5. Wing aspect ratio (WAR) for species available in[39] and mean Kipp index for species ringed in Tabarca that had available data for its calculation. CorrRDA1 and CorrRDA2 identify species with a significant negative correlation with the first two axes of RDA analysis (see Table 4). Area and MinDSouthLand identify species with a significant positive effect of these variables on their abundance index according to GLMM presented in Table S3. *: Kipp index calculated with data from a nearby ringing station on the mainland (Hondo Natural Park).

Species	Acronym	WAR	Kipp index	SD	N	CorrRDA1	CorrRDA2	Area	MinDSouthLand
Acrocephalus arundinaceus	ACRARU*	4.51	28.17	1.71	78	-			
Acrocephalus schoenobaenus	ACRSCH	4.69	27.42	0.84	3	-	-		
Acrocephalus scirpaceus	ACRSCI	4.39	25.61	2.83	5		-		+
Anthus trivialis	ANTTRI	4.79	28.66	1.06	2	-		+	+
Calandrella brachydactyla	CALBRA		35.11		1	-		+	+
Emberiza hortulana	EMBHOR	4.28	28.73		1		-		
Ficedula albicollis	FICALB	4.96				-			
Ficedula hypoleuca	FICHYP	4.67	30.48	2.93	28	-		+	
Hippolais icterina	HIPICT	4.57	29.57	2.93	2	-			
Hippolais polyglotta	HIPPOL		24.31	2.24	53		-		
Iduna opaca	IDUOPA	3.99	24.07	2.04	3		-		+
Lanius collurio	LANCOL	4.87							
Lanius senator	LANSEN		29.69	3.76	42	-	-	+	
Locustella naevia	LOCNAE	4.37							+
Luscinia megarhynchos	LUSMEG	4.60	26.76	2.78	67		-		+
Motacilla flava	MOTFLA	4.71							+
Muscicapa striata	MUSSTR	4.81	31.34	1.55	16	-	-		
Oenanthe hispanica	OENHIS		24.48	2.28	7		-		
Oenanthe oenanthe	OENOEN	4.72	31.01	3.85	116		-		
Oriolus oriolus	ORIORI	5.27	38.56		1	-		+	
Phoenicurus phoenicurus	PHOPHO	4.36	26.36	2.50	215	-	-		+
Phylloscopus bonelli	PHYBON		22.59	2.30	29				
Phylloscopus trochilus	PHYLUS	4.28	26.49	2.81	795		-		+
Phylloscopus sibilatrix	PHYSIB*	4.81	30.63		1	-		+	
Saxicola rubetra	SAXRUB	4.68	28.88	4.08	10	-		+	+
Sylvia borin	SYLBOR	4.86	30.10	2.26	28	-	-	+	
Sylvia cantillans	SYLCAN		21.13	2.88	112		-		+
Sylvia communis	SYLCOM	4.24	24.12	2.44	87	-	-	+	+
Sylvia curruca	SYLCUR	4.06							
Sylvia hortensis	SYLHOR		22.36	1.89	11		-		+

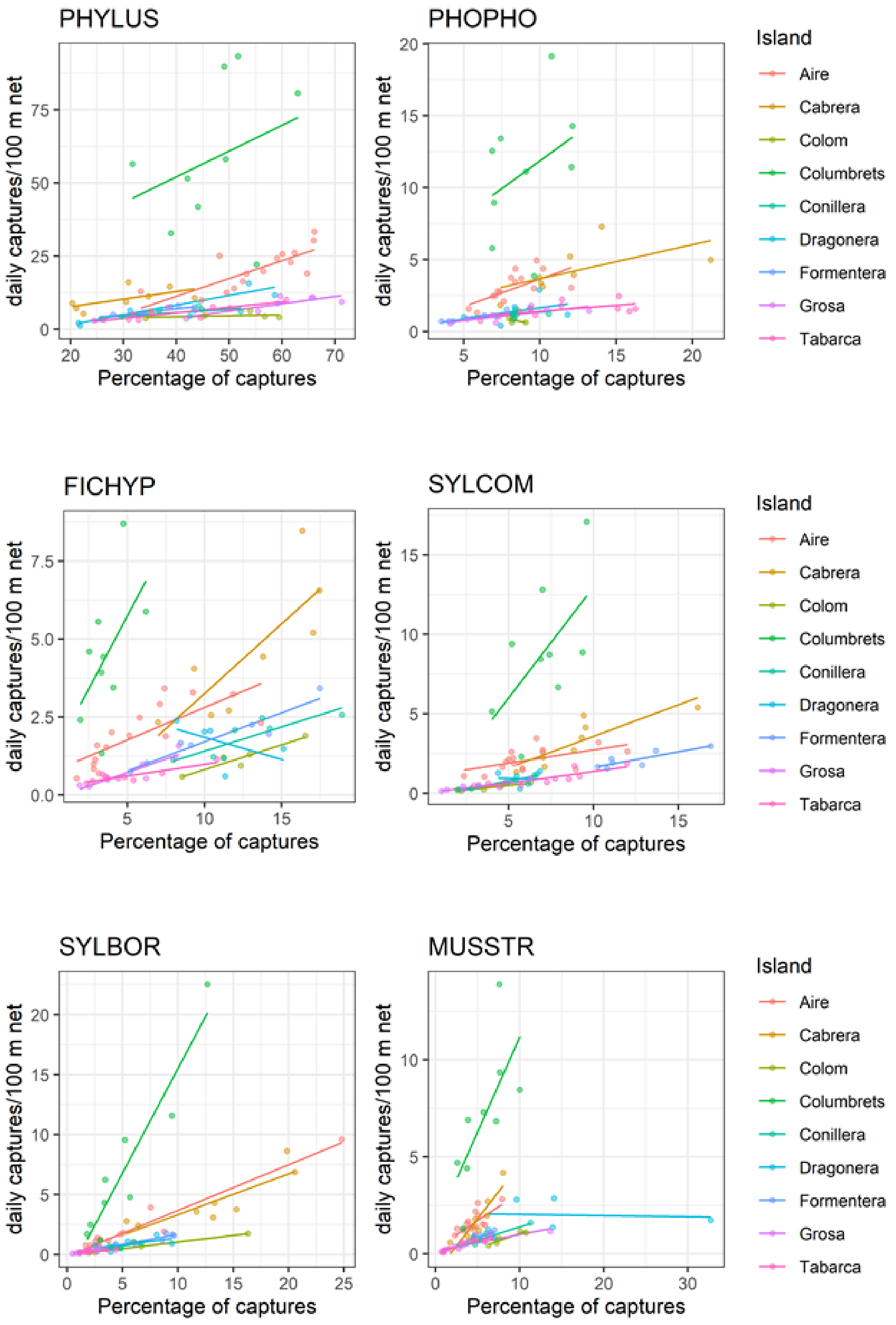

Figure S1. Relationship between percentage of captures and mean daily number of captures per 100 m of mist net of most ringed species in study islands. Each point corresponds to a spring migration season. An independent linear regression has been fitted for each island.

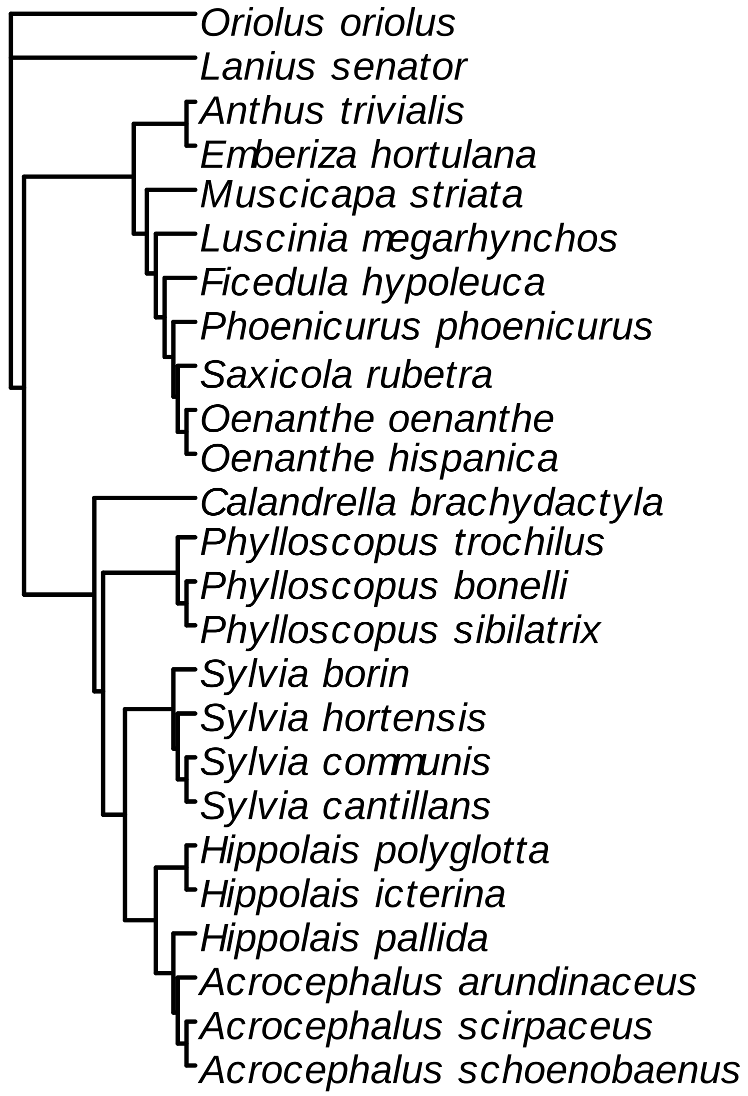

Figure S2. Consensus tree for the set of species with Kipp index estimate available.

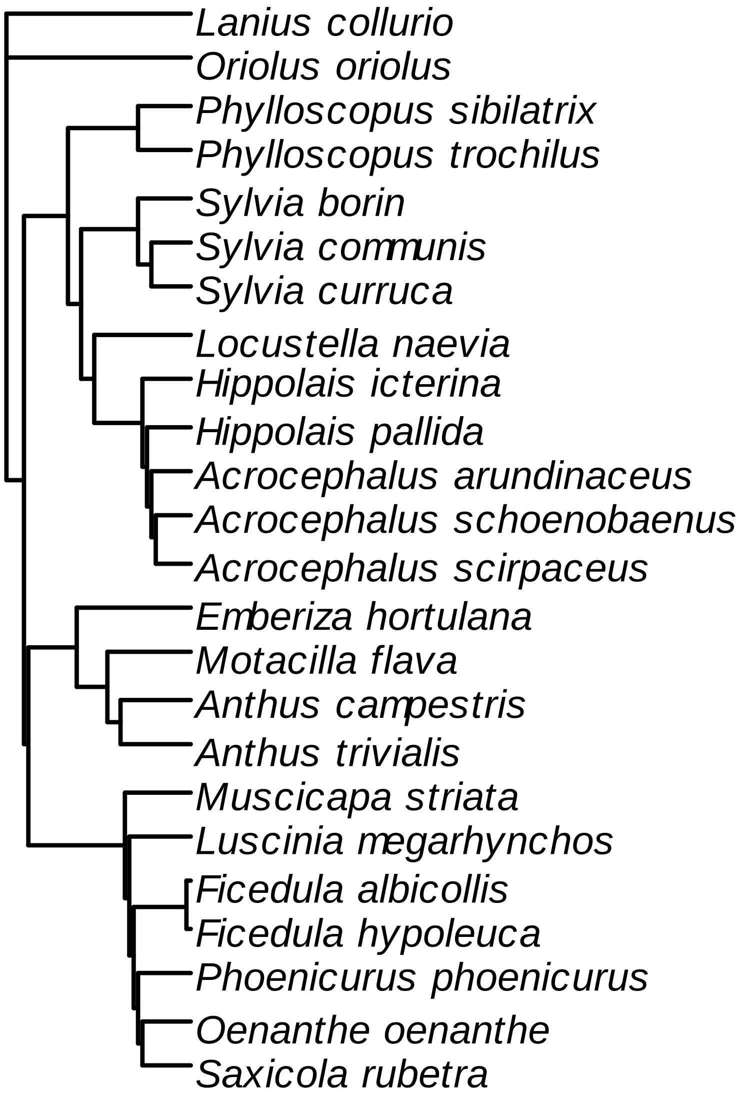

Figure S3. Consensus tree for the set of species with Wing Aspect Ratio (WAR) estimate available.

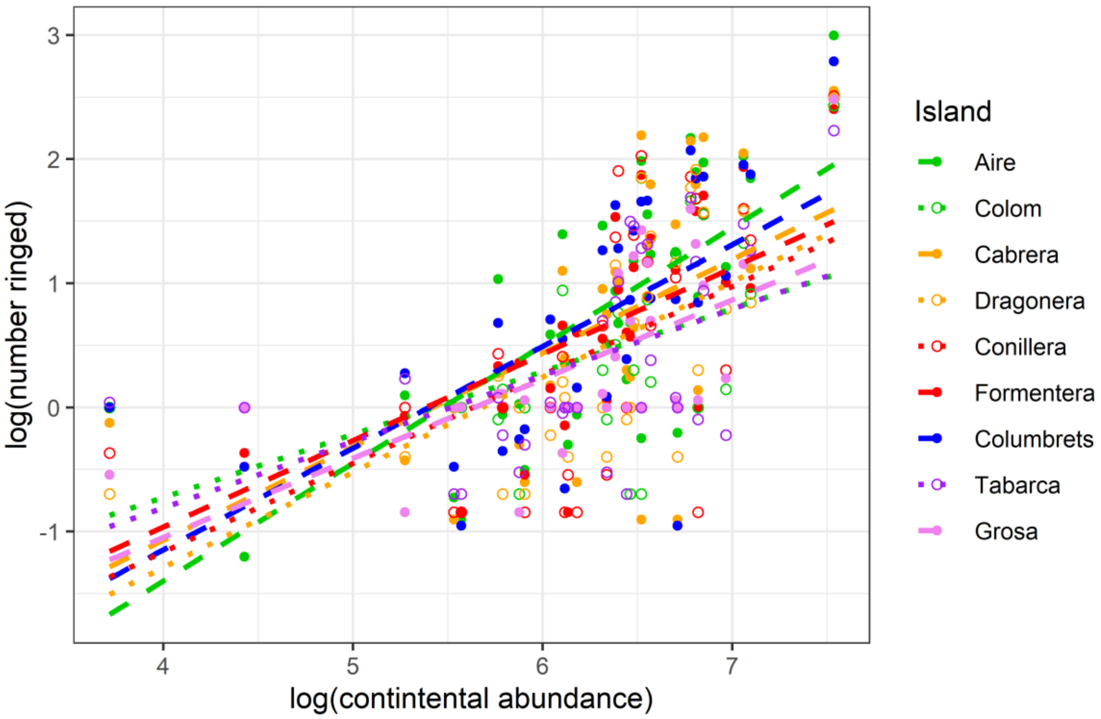

Figure S4. Relationship between the continental abundance of each species estimated for the 27 members of the European Union (see Table S1) and the total number ringed on each island (both variables log-transformed). A linear regression was fitted for each island but slopes are not different between islands. Data and regression lines for pairs of islands located at similar longitude are depicted with the same color. Within each pair the island with a more southern position is represented with a filled circle and the island in the northern position with a hollow circle.

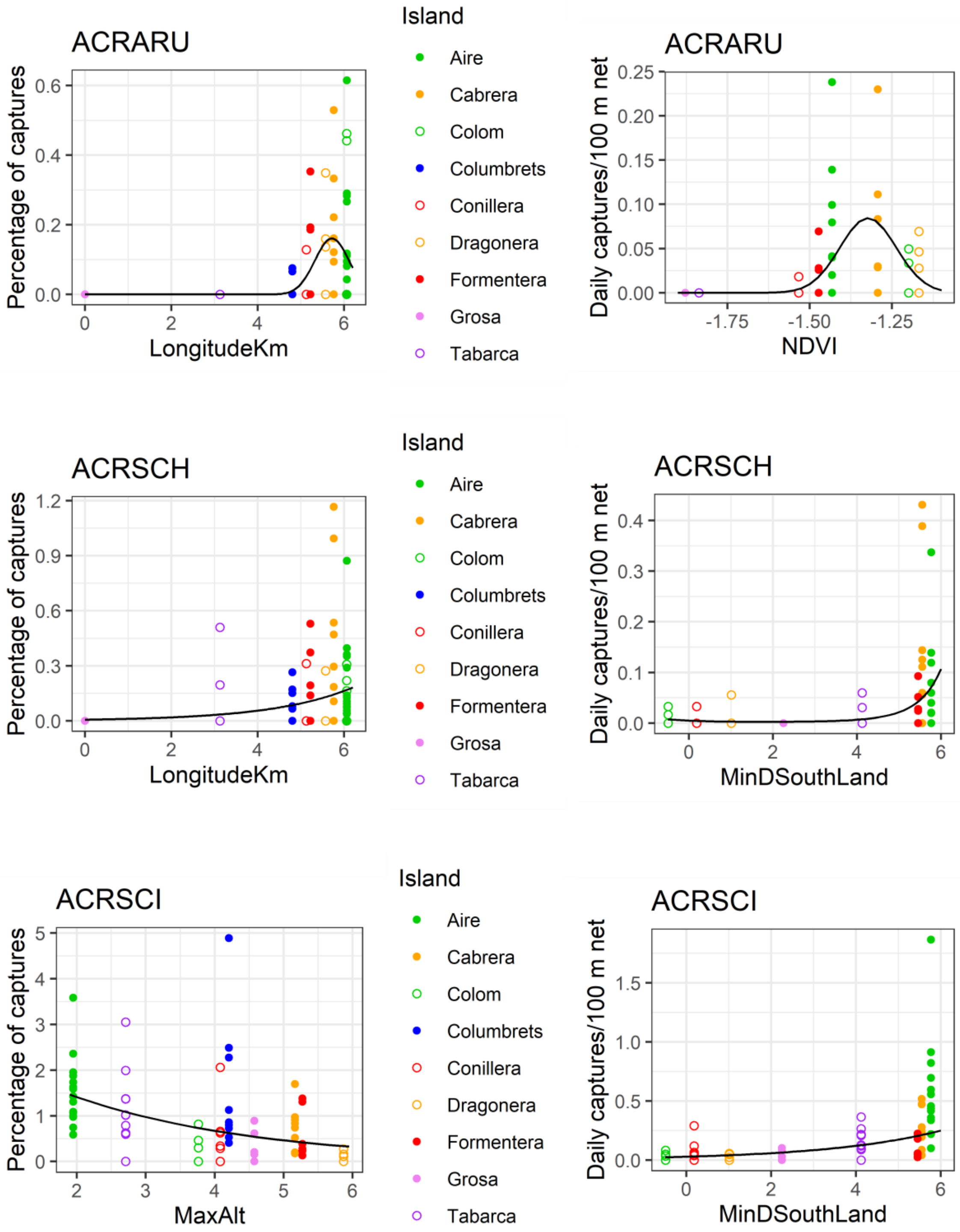

ACRARU
Percentage of captures
0.0
0.2
0.4
0.6
0
2
4
6
LongitudeKm
Island
Aire
Cabrera
Colom
Columbrets
Conillera
Dragonera
Formentera
Grosa
Tabarca
ACRARU
Daily captures/100 m net
0.00
0.05
0.10
0.15
0.20
0.25
-1.75
-1.50
-1.25
NDVI
ACRSCH
Percentage of captures
0.0
0.3
0.6
0.9
1.2
0
2
4
6
LongitudeKm
Island
Aire
Cabrera
Colom
Columbrets
Conillera
Dragonera
Formentera
Grosa
Tabarca
ACRSCH
Daily captures/100 m net
0.0
0.1
0.2
0.3
0.4
0
2
4
6
MinDSouthLand
ACRSCI
Percentage of captures
0
1
2
3
4
5
2
3
4
5
6
MaxAlt
Island
Aire
Cabrera
Colom
Columbrets
Conillera
Dragonera
Formentera
Grosa
Tabarca
ACRSCI
Daily captures/100 m net
0.0
0.5
1.0
1.5
0
2
4
6
MinDSouthLand

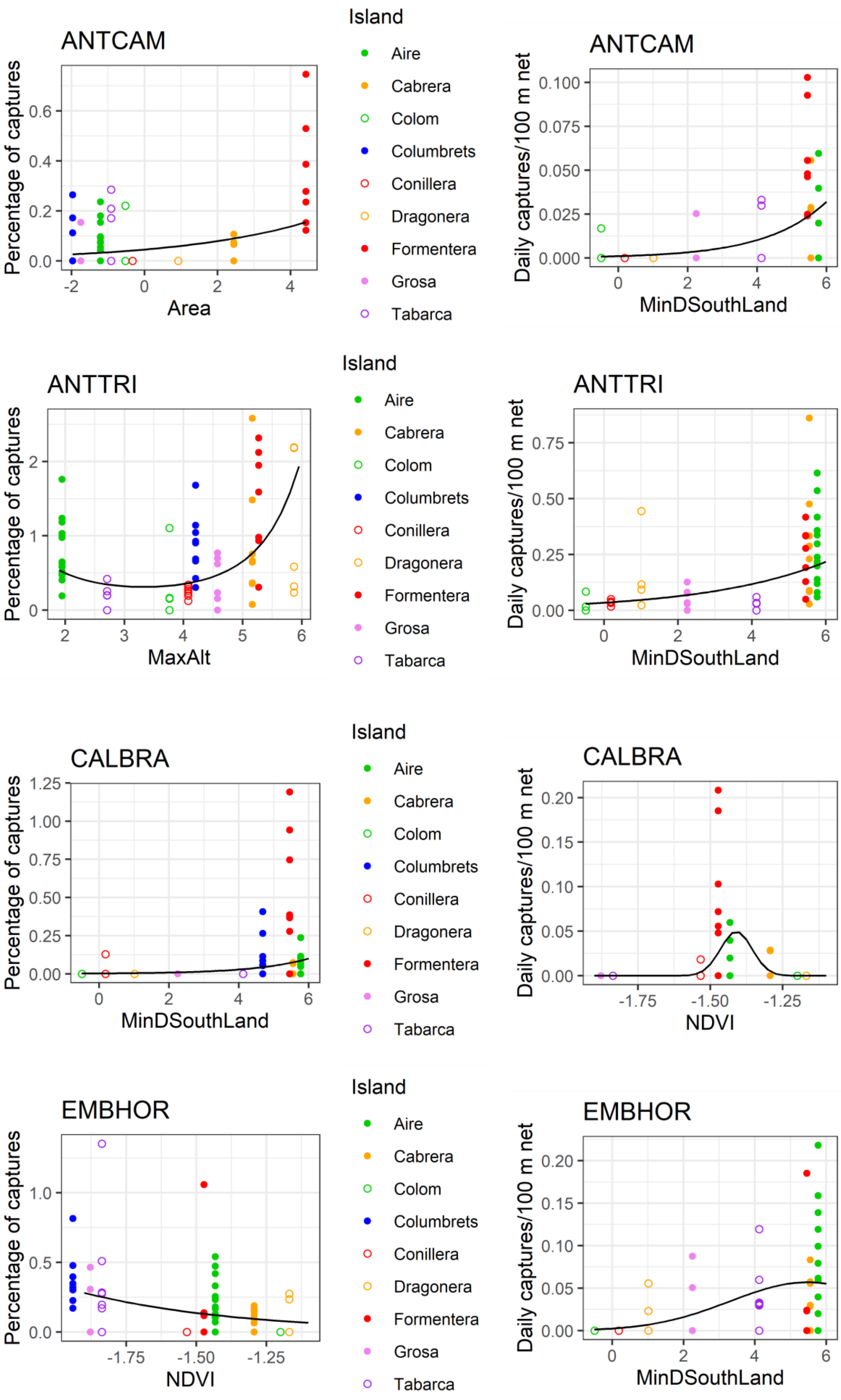

ANTCAM
Percentage of captures
Area
Island
Aire
Cabrera
Colom
Columbrets
Conillera
Dragonera
Formentera
Grosa
Tabarca
ANTCAM
Daily captures/100 m net
MinDSouthLand
ANTTRI
Percentage of captures
MaxAlt
ANTTRI
Daily captures/100 m net
MinDSouthLand
CALBRA
Percentage of captures
MinDSouthLand
CALBRA
Daily captures/100 m net
NDVI
EMBHOR
Percentage of captures
NDVI
EMBHOR
Daily captures/100 m net
MinDSouthLand

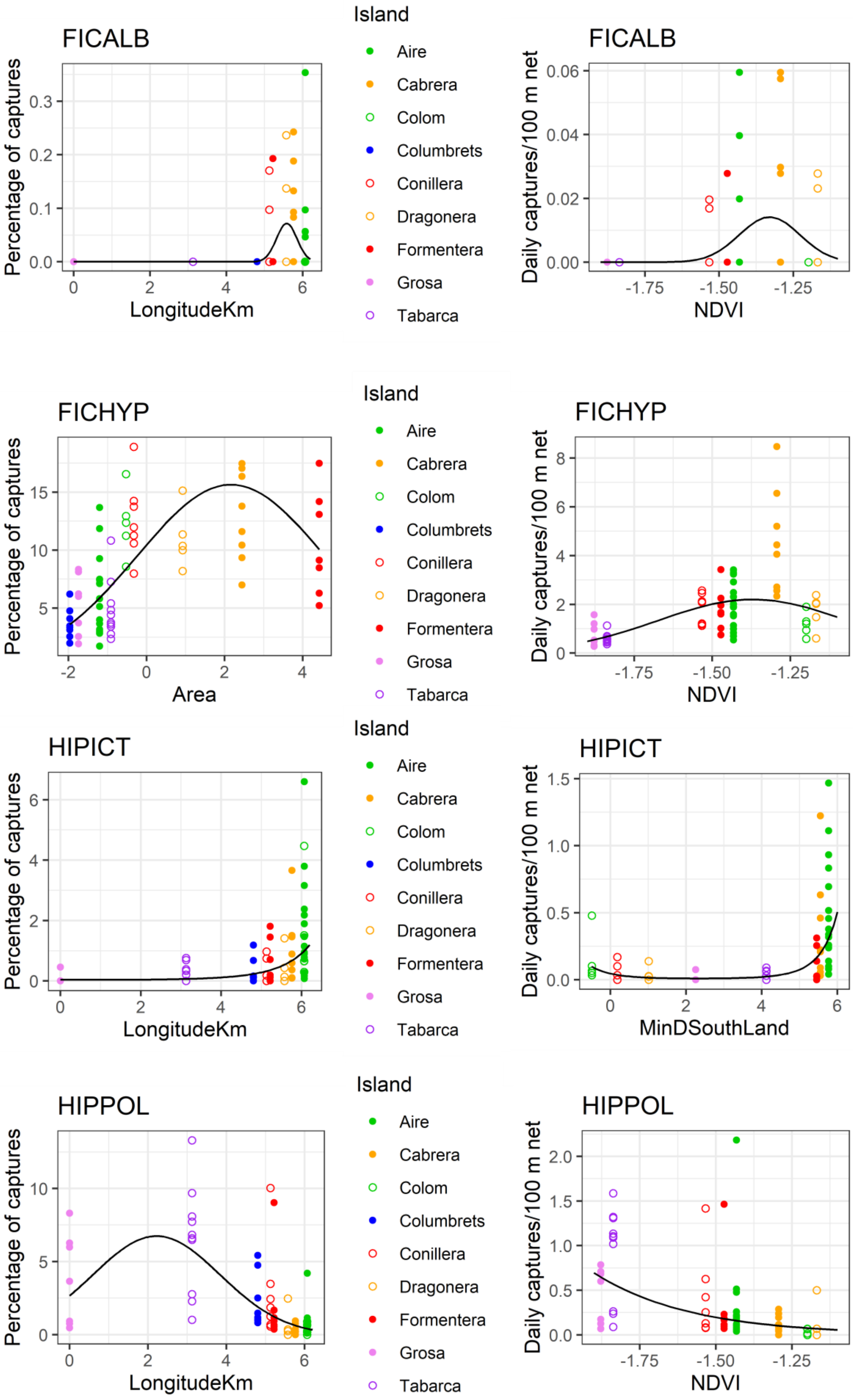
FICALB
Percentage of captures
LongitudeKm
Island
Aire
Cabrera
Colom
Columbrets
Conillera
Dragonera
Formentera
Grosa
Tabarca
FICALB
Daily captures/100 m net
NDVI
FICHYP
Percentage of captures
Area
FICHYP
Daily captures/100 m net
NDVI
HIPICT
Percentage of captures
LongitudeKm
HIPICT
Daily captures/100 m net
MinDSouthLand
HIPPOL
Percentage of captures
LongitudeKm
HIPPOL
Daily captures/100 m net
NDVI

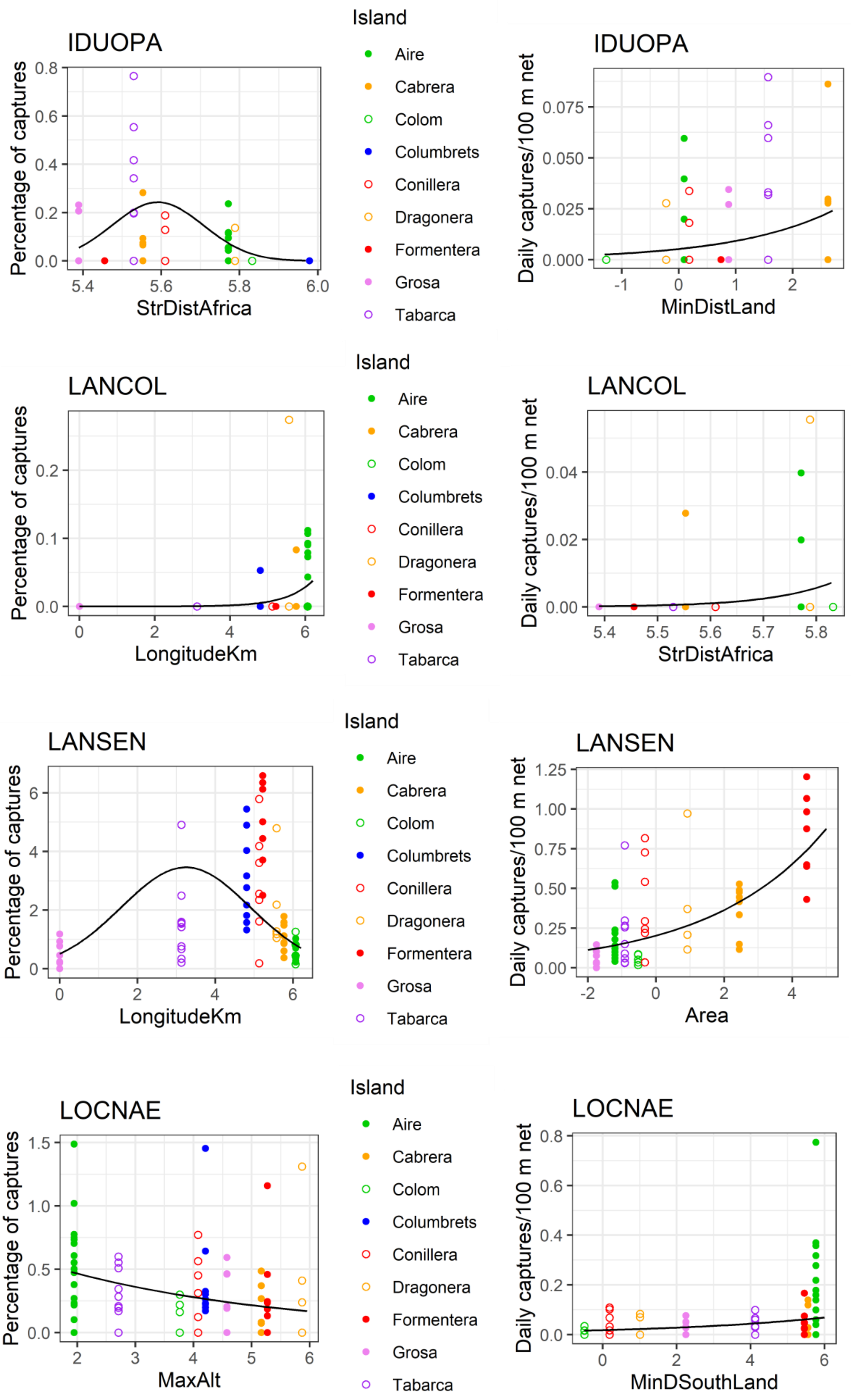
IDUOPA
Percentage of captures
StrDistAfrica
Island
Aire
Cabrera
Colom
Columbrets
Conillera
Dragonera
Formentera
Grosa
Tabarca
IDUOPA
Daily captures/100 m net
MinDistLand
LANCOL
Percentage of captures
LongitudeKm
LANCOL
Daily captures/100 m net
StrDistAfrica
LANSEN
Percentage of captures
LongitudeKm
LANSEN
Daily captures/100 m net
Area
LOCNAE
Percentage of captures
MaxAlt
LOCNAE
Daily captures/100 m net
MinDSouthLand

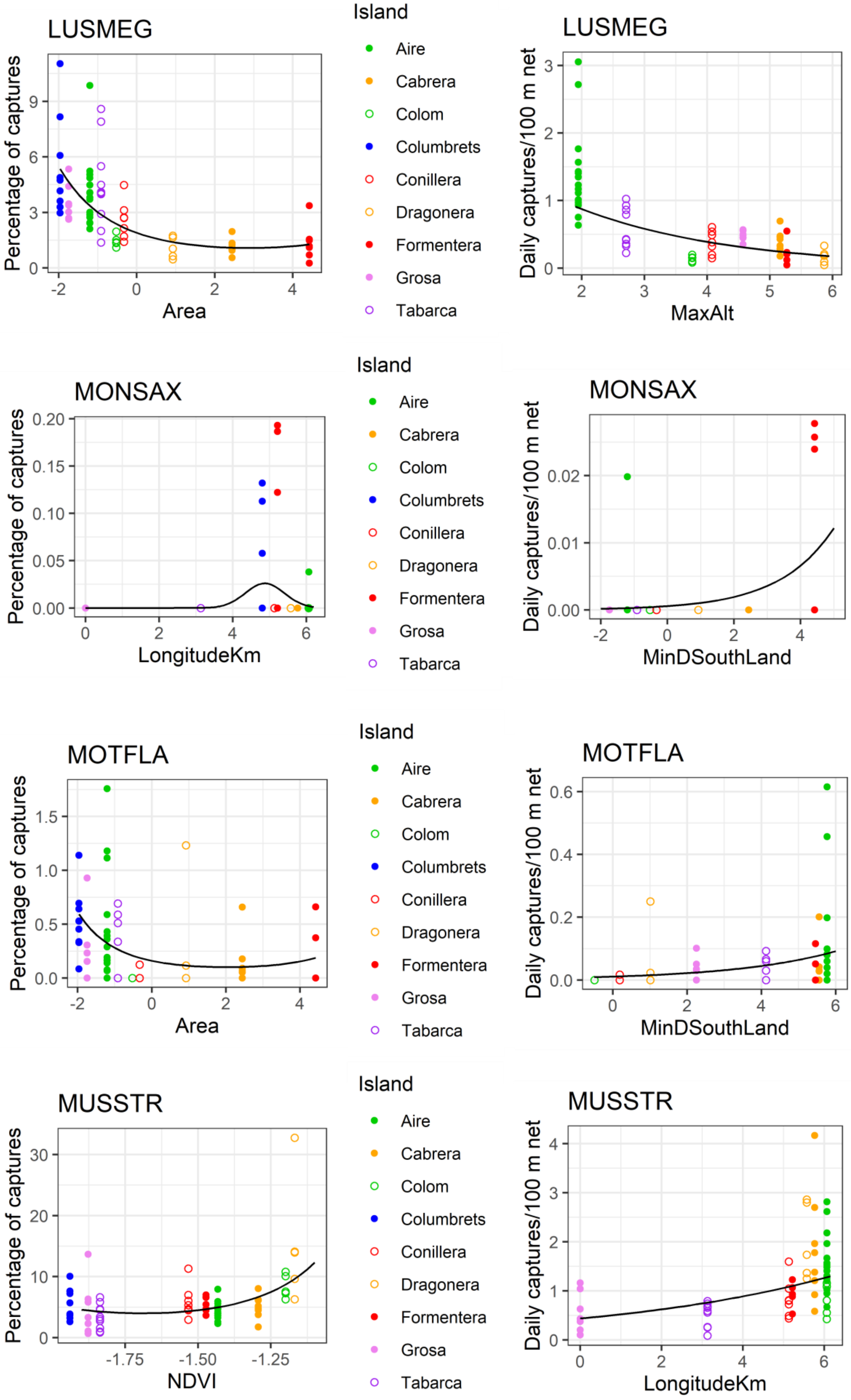

LUSMEG
Percentage of captures
Area
Island
Aire
Cabrera
Colom
Columbrets
Conillera
Dragonera
Formentera
Grosa
Tabarca
LUSMEG
Daily captures/100 m net
MaxAlt
MONSAX
Percentage of captures
LongitudeKm
MONSAX
Daily captures/100 m net
MinDSouthLand
MOTFLA
Percentage of captures
Area
MOTFLA
Daily captures/100 m net
MinDSouthLand
MUSSTR
Percentage of captures
NDVI
MUSSTR
Daily captures/100 m net
LongitudeKm

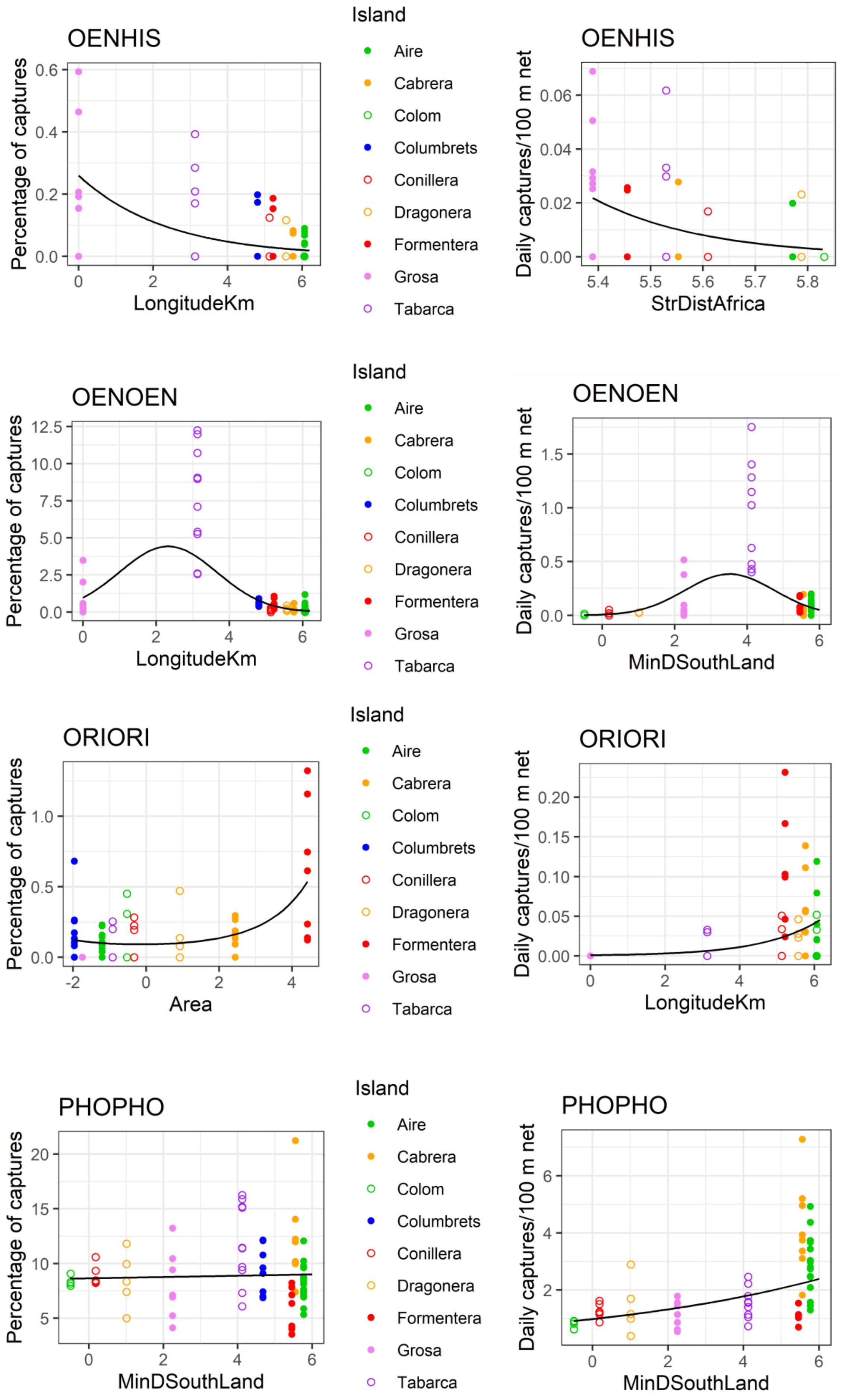
OENHIS
Percentage of captures
0.6
0.4
0.2
0.0
0
2
4
6
LongitudeKm
Island
Aire
Cabrera
Colom
Columbrets
Conillera
Dragonera
Formentera
Grosa
Tabarca
OENHIS
Daily captures/100 m net
0.06
0.04
0.02
0.00
5.4
5.5
5.6
5.7
5.8
StrDistAfrica
OENOEN
Percentage of captures
12.5
10.0
7.5
5.0
2.5
0.0
0
2
4
6
LongitudeKm
Island
Aire
Cabrera
Colom
Columbrets
Conillera
Dragonera
Formentera
Grosa
Tabarca
OENOEN
Daily captures/100 m net
1.5
1.0
0.5
0.0
0
2
4
6
MinDSouthLand
ORIORI
Percentage of captures
1.0
0.5
0.0
-2
0
2
4
Area
Island
Aire
Cabrera
Colom
Columbrets
Conillera
Dragonera
Formentera
Grosa
Tabarca
ORIORI
Daily captures/100 m net
0.20
0.15
0.10
0.05
0.00
0
2
4
6
LongitudeKm
PHOPHO
Percentage of captures
20
15
10
5
0
2
4
6
MinDSouthLand
Island
Aire
Cabrera
Colom
Columbrets
Conillera
Dragonera
Formentera
Grosa
Tabarca
PHOPHO
Daily captures/100 m net
6
4
2
0
2
4
6
MinDSouthLand

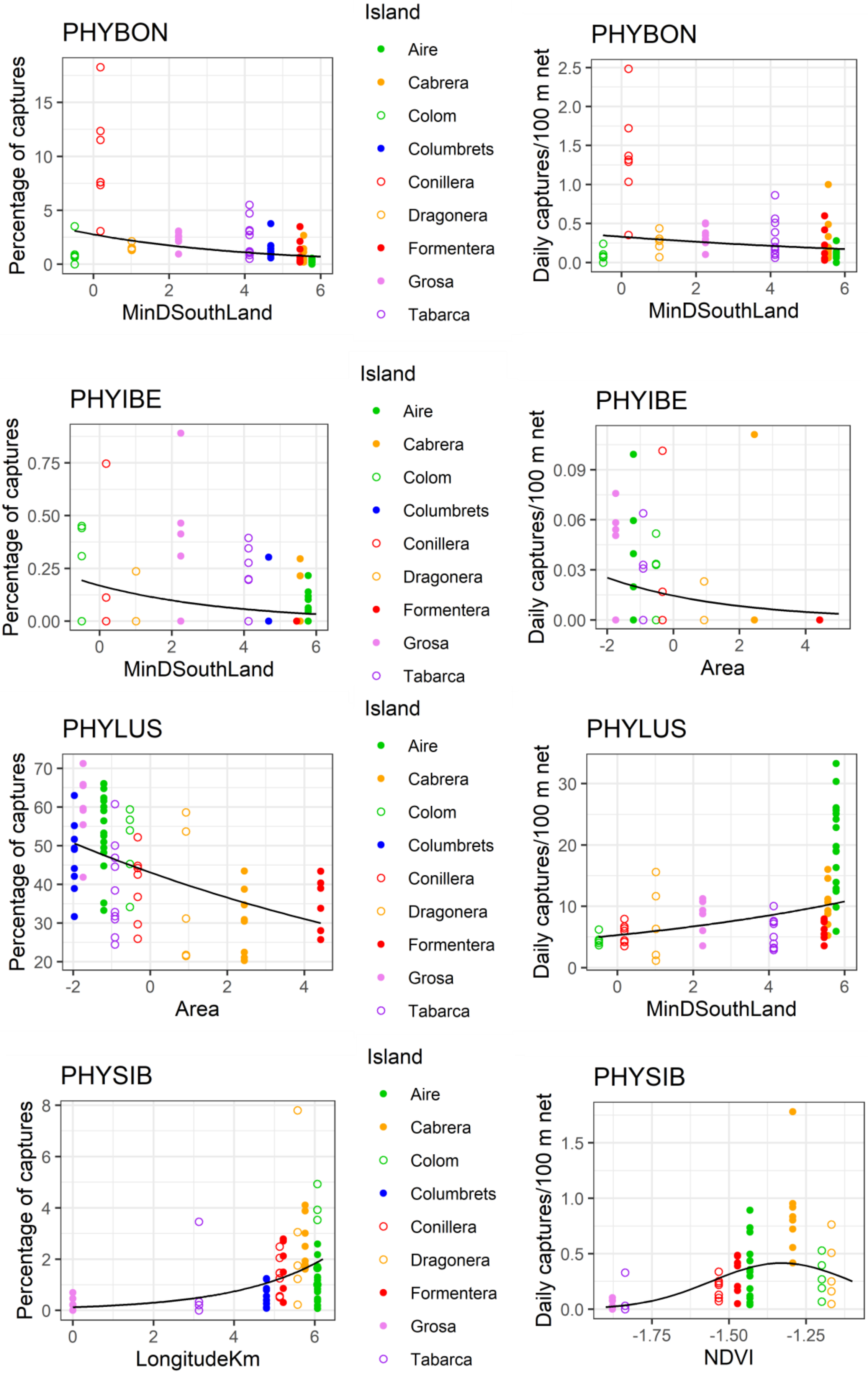
PHYBON
Percentage of captures
MinDSouthLand
Island
Aire
Cabrera
Colom
Columbrets
Conillera
Dragonera
Formentera
Grosa
Tabarca
PHYBON
Daily captures/100 m net
MinDSouthLand
PHYIBE
Percentage of captures
MinDSouthLand
Island
Aire
Cabrera
Colom
Columbrets
Conillera
Dragonera
Formentera
Grosa
Tabarca
PHYIBE
Daily captures/100 m net
Area
PHYLUS
Percentage of captures
Area
Island
Aire
Cabrera
Colom
Columbrets
Conillera
Dragonera
Formentera
Grosa
Tabarca
PHYLUS
Daily captures/100 m net
MinDSouthLand
PHYSIB
Percentage of captures
LongitudeKm
Island
Aire
Cabrera
Colom
Columbrets
Conillera
Dragonera
Formentera
Grosa
Tabarca
PHYSIB
Daily captures/100 m net
NDVI

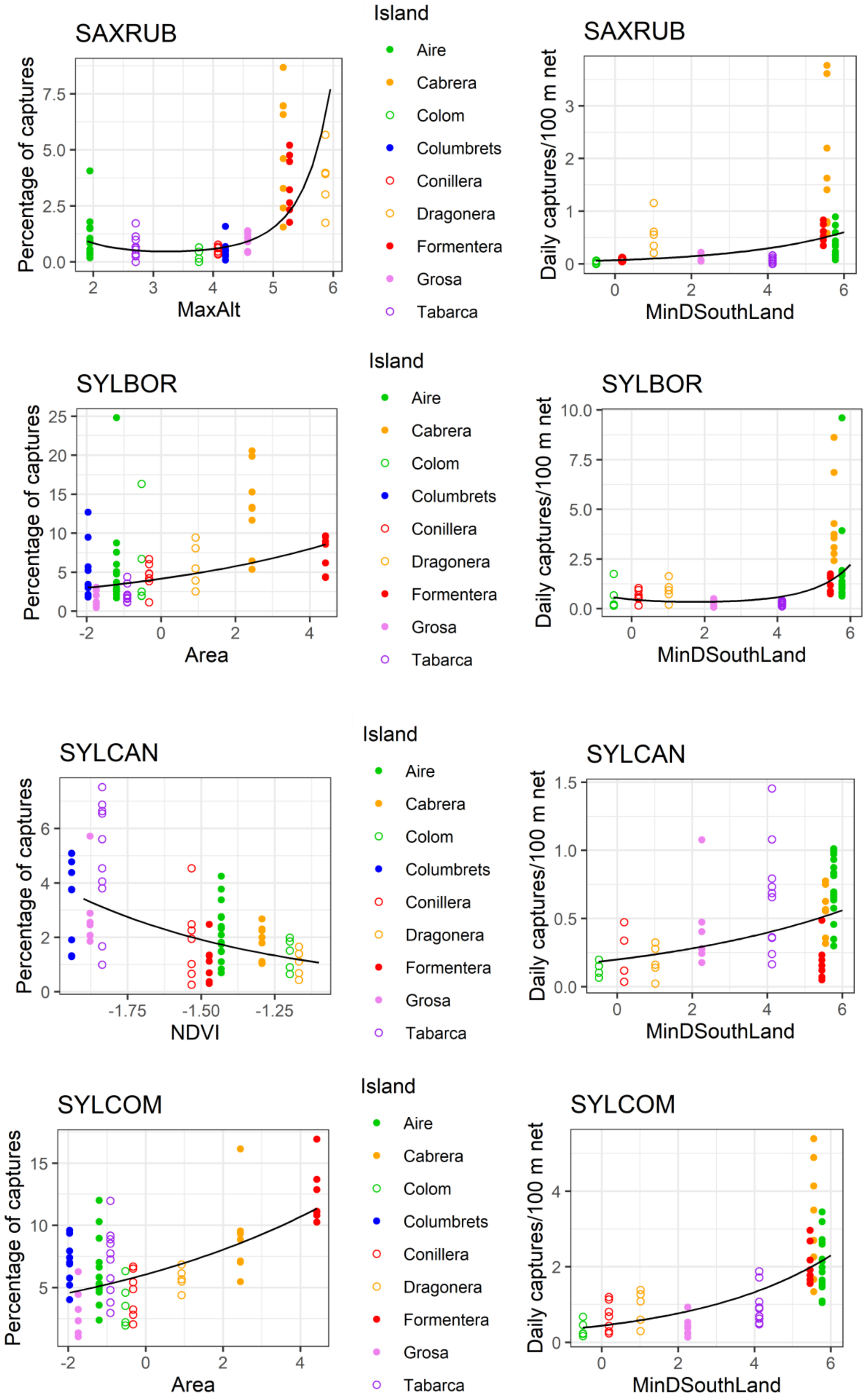

SAXRUB
Percentage of captures
MaxAlt
Island
Aire
Cabrera
Colom
Columbrets
Conillera
Dragonera
Formentera
Grosa
Tabarca
SAXRUB
Daily captures/100 m net
MinDSouthLand
SYLBOR
Percentage of captures
Area
Island
Aire
Cabrera
Colom
Columbrets
Conillera
Dragonera
Formentera
Grosa
Tabarca
SYLBOR
Daily captures/100 m net
MinDSouthLand
SYLCAN
Percentage of captures
NDVI
Island
Aire
Cabrera
Colom
Columbrets
Conillera
Dragonera
Formentera
Grosa
Tabarca
SYLCAN
Daily captures/100 m net
MinDSouthLand
SYLCOM
Percentage of captures
Area
Island
Aire
Cabrera
Colom
Columbrets
Conillera
Dragonera
Formentera
Grosa
Tabarca
SYLCOM
Daily captures/100 m net
MinDSouthLand

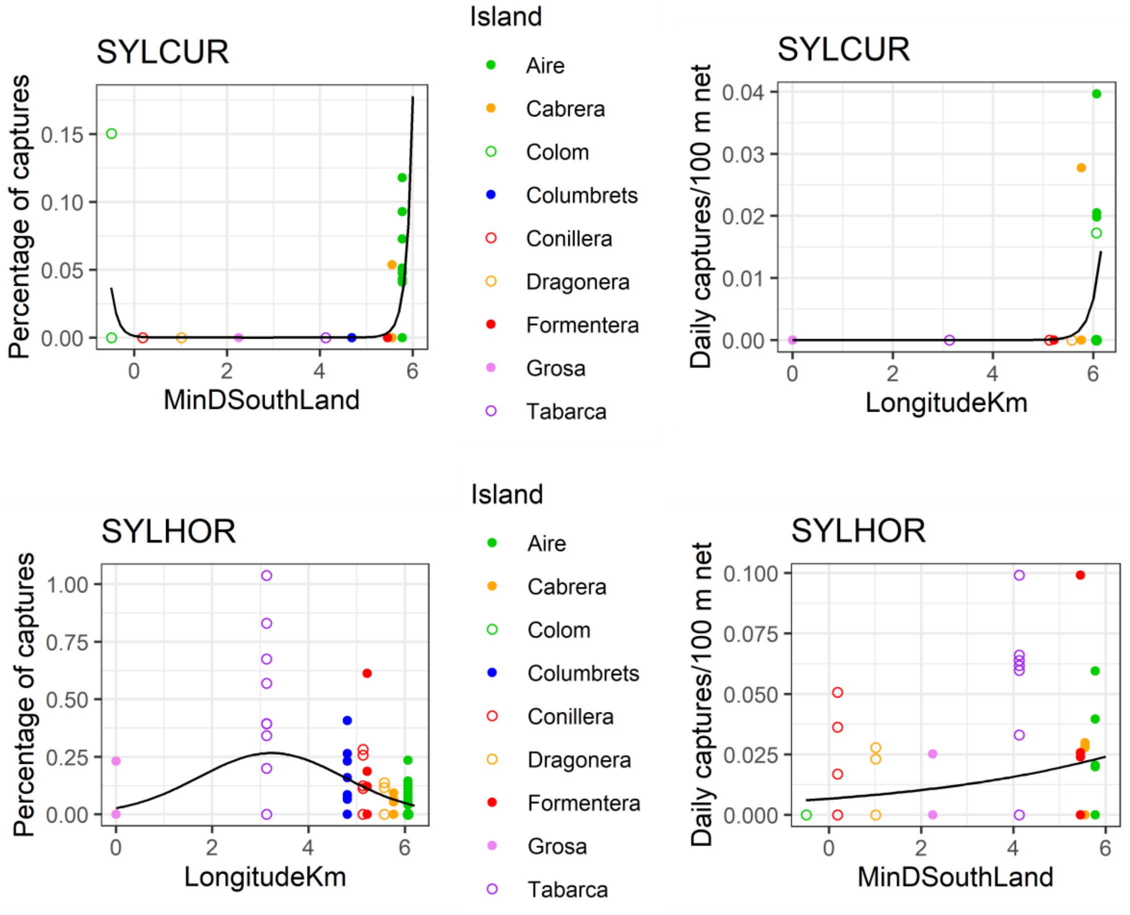

Figure S5. Best models adjusted to percentage of captures (left) and daily captures for 100 m of mist net (right) for all selected species. Predictor variables are log-transformed. In species in which the best model included the same predictor in both cases, the second-best model is shown for the dependent variable with the lowest ΔAICc of the second model, in order to avoid redundant information. In two species, PHOPHO and PHYBON, it was only possible to fit a model for the number of captures and percentage of captures, respectively. In these species, the same predictor was used in their graphics. Pairs of islands located at similar longitude are depicted with the same color. Within each pair the island with a more southern position is represented with a filled circle and the island in the northern position with a hollow circle.
